# A Förster Resonance Energy Transfer (FRET)-based System Provides Insight into the Ordered Assembly of Yeast Septin Hetero-octamers*[Fn FN2]

**DOI:** 10.1074/jbc.M115.683128

**Published:** 2015-09-28

**Authors:** Elizabeth A. Booth, Eleanor W. Vane, Dustin Dovala, Jeremy Thorner

**Affiliations:** From the ‡ Division of Biochemistry, Biophysics, and Structural Biology, Department of Molecular and Cell Biology, University of California, Berkeley, California 94720-3202 and; the § Program in Microbial Pathogenesis and Host Defense, Department of Microbiology and Immunology, University of California School of Medicine, San Francisco, California 94158-2200

**Keywords:** fluorescence, protein engineering, protein purification, protein self-assembly, Saccharomyces cerevisiae

## Abstract

Prior studies in both budding yeast (*Saccharomyces cerevisiae*) and in human cells have established that septin protomers assemble into linear hetero-octameric rods with 2-fold rotational symmetry. In mitotically growing yeast cells, five septin subunits are expressed (Cdc3, Cdc10, Cdc11, Cdc12, and Shs1) and assemble into two types of rods that differ only in their terminal subunit: Cdc11-Cdc12-Cdc3-Cdc10-Cdc10-Cdc3-Cdc12-Cdc11 and Shs1-Cdc12-Cdc3-Cdc10-Cdc10-Cdc3-Cdc12-Shs1. EM analysis has shown that, under low salt conditions, the Cdc11-capped rods polymerize end to end to form long paired filaments, whereas Shs1-capped rods form arcs, spirals, and rings. To develop a facile method to study septin polymerization *in vitro*, we exploited our previous work in which we generated septin complexes in which all endogenous cysteine (Cys) residues were eliminated by site-directed mutagenesis, except an introduced E294C mutation in Cdc11 in these experiments. Mixing samples of a preparation of such single-Cys containing Cdc11-capped rods that have been separately derivatized with organic dyes that serve as donor and acceptor, respectively, for FRET provided a spectroscopic method to monitor filament assembly mediated by Cdc11-Cdc11 interaction and to measure its affinity under specified conditions. Modifications of this same FRET scheme also allow us to assess whether Shs1-capped rods are capable of end to end association either with themselves or with Cdc11-capped rods. This FRET approach also was used to follow the binding to septin filaments of a septin-interacting protein, the type II myosin-binding protein Bni5.

## Teaser

**Background:** Septins self-assemble into hetero-octameric rods and higher order structures and recruit other proteins.

**Results:** A spectroscopic method (FRET) to measure septin interactions and binding of associated proteins was developed.

**Conclusion:** End to end polymerization of Cdc11-capped rods, heterotypic end to end junctions between Cdc11-capped rods and Shs1-capped rods, and binding of an associated protein were demonstrated.

**Significance:** This spectroscopic assay provides new insights about these polymeric proteins.

## Introduction

Septins are a family of GTP-binding proteins found in all eukaryotes (except plants) (1, 2) and have roles in cytokinesis, cell compartmentation, and membrane remodeling (3–5). The *Saccharomyces cerevisiae* genome encodes five septins that are expressed in mitotically growing cells: Cdc3, Cdc10, Cdc11, Cdc12, and Shs1 (6, 7). Four of these proteins were first identified because the corresponding loci were among the temperature-sensitive cell division cycle (*cdc*) mutations isolated by Hartwell *et al.* (8, 9) and specifically exhibited failure of cytokinesis at the restrictive temperature. The fifth member was identified later by other means and recognized as a gene product homologous to the other four (10, 11).

During initial characterization of the *cdc* mutants by EM, it was noted that, after incubation of cells at the restrictive temperature, the *cdc3*, *cdc10*, *cdc11*, and *cdc12* mutants uniquely lost filament-like striations found at the bud neck in control cells (12). These striations were 10 nm wide and ∼28 nm apart. Indirect immunofluorescence with anti-septin antibodies (13–15) and, later, fusion of septins to fluorescent proteins (16, 17) demonstrated that septins are located at and likely constituents of the filamentous bud neck structure. Subsequently, purification of septins from yeast (18) and as recombinant proteins from bacteria (19–21) showed that Cdc3, Cdc10, Cdc11, and Cdc12 were sufficient to form long paired filaments *in vitro* (22) that closely resemble those seen by EM at bud neck *in vivo* (12, 23, 24).

Ensuing work showed that the five mitotic septins of yeast form two types of linear, apolar hetero-octameric complexes of a defined order, which differ only with respect to the terminal subunit present: Cdc11-Cdc12-Cdc3-Cdc10-Cdc10-Cdc3-Cdc12-Cdc11 and Shs1-Cdc12-Cdc3-Cdc10-Cdc10-Cdc3-Cdc12-Shs1 (22, 25). These two types of rods are very stable, even in high salt (≥250 mm) buffers. When the salt concentration is reduced (<100 mm), Cdc11-capped rods polymerize end to end into long paired filaments, as visualized *in vitro* by EM (22) and by super-resolution fluorescence microscopy (26). By contrast, Shs1-capped rods associate laterally in a staggered manner generating bundles that interact to form arcs, spirals, and rings (25). The septin collar at the bud neck appears to have three primary functions: (i) it establishes a cortical diffusion barrier (27, 28); (ii) it serves as a scaffold to recruit other proteins (29, 30); and (iii) it promotes membrane curvature either directly by deforming the membrane and/or indirectly by recruiting other proteins that can remodel membranes (31–33).

**FIGURE 1.**
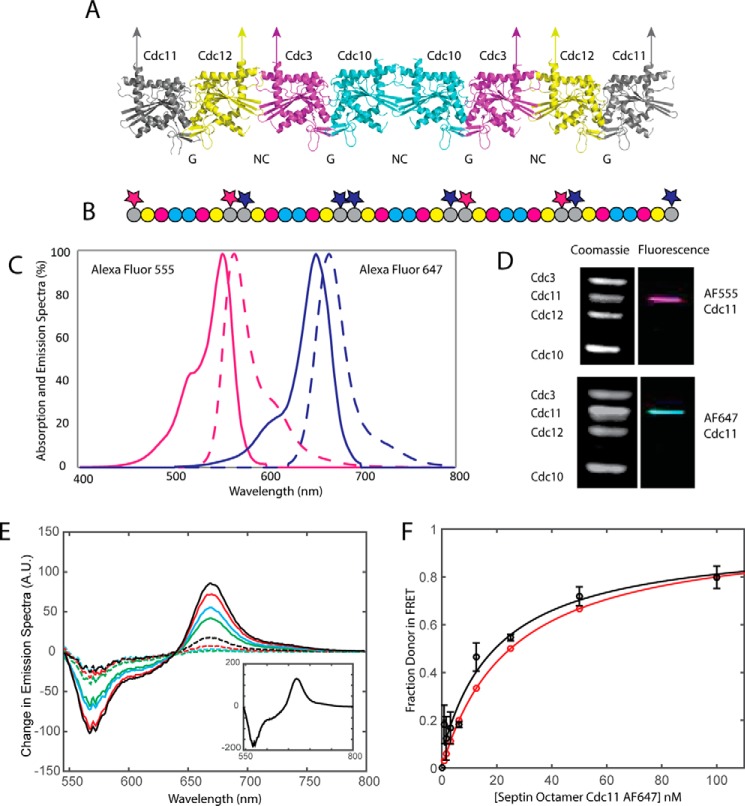
**Labeling terminal septin subunit (Cdc11) allows for establishment of a FRET system.** *A*, schematic representation of subunits within the yeast septin octamer as predicted by Phyre II and aligned to the crystal structure of the human septin hexamer (2QAG) in PyMOL. *B*, schematic showing possible arrangements of Cdc11-labeled septin subunits. Donor- and acceptor-labeled septins are allowed to assemble stochastically. *C*, normalized excitation and emission spectra as measured of AF555 and AF647. *D*, Coomassie gels and typhoon scans of Cdc11 septin octamers labeled at the terminal septin with AF555 and AF647. *E*, FRET emission spectra with 25 nm Cdc11^AF555^-labeled octamers recorded at increasing concentrations of Cdc11^AF647^-labeled octamers: 0.78 nm (*dashed green*), 1.56 nm (*dashed cyan*), 3.13 nm (*dashed red*), 6.25 nm (*dashed black*), 12.5 nm (*solid green*), 25 nm (*solid cyan*), 50 nm (*solid red*), and 100 nm (*solid black*). The *inset* illustrates the result of the PCA. *F*, binding curve corresponding to fluorescence spectra in *E*. The *black circles* with S.E. *error bars* correspond to experimental data. The *black line* shows the fitted binding curve. The *red curve* is the predicted binding curve from stochastic assembly of the septin polymeric complex.

As for yeast, members of the family of 13 human septins also form linear hetero-octameric rods, the most abundant of which has the composition Sept9-Sept7-Sept6-Sept2-Sept2-Sept6-Sept7-Sept9 (34, 35). Moreover, crystal structures of individual human septins (36, 37) or septins from other animal cells (38) and of a human hetero-hexameric complex (39, 40) revealed the nature of the two alternating interfaces that mediate linear assembly of the protomers into the hetero-oligomeric rod. The G interface between two subunits is an *en face* interaction that involves residues in and around the GTP-binding pockets of each protomer, and at the opposing surface (180° away from the G interface), is the NC interface wherein two subunits associate via contacts provided by residues in and around the N- and C-terminal segments of each protomer (39). Biochemical analysis (19, 41) and structural studies (38, 40) have shown how GTP binding, and, in the case of certain septin subunits, GTP hydrolysis, influences subunit conformation and interaction. In the yeast hetero-octamer, the Cdc11-Cdc12 interface is a G interface, the Cdc12-Cdc3 interface is an NC interface, and so forth (see Fig. 1*A*). Aside from their globular GTP-binding fold, the yeast mitotic septins have N-terminal extensions of variable length, with Cdc11 the shortest (12 residues) and Cdc3 the longest (110 residues), and, with the exception of Cdc10, also possess a long (≥100 residue) flexible C-terminal extension (CTE)^5^ that contains a segment (∼40 residues) with a strongly predicted propensity for coiled-coil formation (20, 42).

To date, septin function *in vivo* has been studied primarily using genetic methods, and septin properties *in vitro* have been studied largely by examining static structures under the EM. Fluorescence microscopy has been used to visualize septin polymerization, but because of the large dimensions of the fluorophores used, either antibodies for immunostaining (20) or fusions to GFP (43) or to the SNAP tag (44), and the diffraction limit of light, such approaches cannot address the molecular details of the mechanism of septin assembly. Furthermore, given the dimensions of the yeast septin hetero-octamer (4 × 32 nm), even super-resolution fluorescence microscopy applied to yeast septin filaments has merely confirmed what is already known about the order of subunits in the hetero-octamer (26).

To understand other self-assembling biopolymers, such as formation of microtubules from tubulin (45, 46) and of F-actin from G-actin (47, 48), and the interaction of these cytoskeletal elements with other proteins that bind to them, it has been exceedingly valuable to have spectroscopic assays to monitor the state of assembly in real time under conditions that can be readily manipulated. For the purposes of interrogating interactions at the protein-protein level, FRET is especially well suited. FRET allows for rapid measurements and is sensitive to the distance between the donor and acceptor fluorophores at the ≤10 nanometer scale, and the donor-acceptor/quencher interaction provides an unambiguous indication of how two elements within the system under study are associating. Given the hetero-oligomeric nature of septin complexes, we felt that FRET could provide a robust and sensitive means to investigate under a wide range of experimental conditions important and heretofore inaccessible mechanistic details about the dynamics and affinities of the protein-protein interactions involved in assembly and organization of septin structures.

Here we describe our development of a reliable FRET-based system for studying the polymerization of septin complexes into filaments, the effects of ionic strength on septin filament assembly, the interplay between Cdc11- and Shs1-capped hetero-octamers, the effects of mutational perturbation of important structural elements in the protomers on their ability to assemble, and the binding of a septin-associated protein. Our findings have important implications for understanding the physiological roles of alternate septin subunits and how septins behave as a polymeric system.

## Experimental Procedures

### 

#### 

##### Expression and Purification of Septin Complexes

Septin subunits were co-expressed from two DUET^TM^ vectors (EMD Millipore) with compatible origins of replication essentially as described previously (20), except that *Escherichia coli* NiCo21 (DE3) (New England Biolabs) was used as the production strain because it has been engineered to prevent recovery of GlmS and several other endogenous *E. coli* Ni^2+^ bead-binding proteins that are normally major contaminants in purifications utilizing IMAC (49). Prior work from this laboratory demonstrated that the Cys-less septins Cdc3(C124V,C253V,C279V), Cdc10(C266S), Cdc11(C43F,C137A,C138A), (His)_6_-Cdc12(C40A,C278S), and Shs1(C29V,C148S) supported yeast cell viability and formed recombinant hetero-octameric complexes that were indistinguishable in their *in vitro* assembly properties from those produced from their wild-type counterparts (50). Using a combination of standard site-directed mutagenesis (51) and sequence- and ligation-independent cloning (52), derivatives of each of the Cys-less septins were constructed in which a single Cys was reinstalled at a selected position. A pACYC plasmid expressing Cdc3(C124V,C253V,C279V) and Cdc11(C42F,C137A,C138A,E294C,Δ303–415) (50) was repaired to restore an intact CTE (residues 303–415) to the *CDC11* ORF. A pACYC plasmid that expresses Cdc3(C124V,C253V,C279V) and Shs1(C29V,C148S,E344C) was constructed for production and purification of Shs1-capped hetero-octamers. Bacterial cultures were grown to an *A*_600 nm_ = 0.8–1.0, induced with isopropyl-β-d-thiogalactoside (final concentration, 0.5 mm) overnight at 16 °C, collected by centrifugation, and resuspended in ice-cold lysis buffer (300 mm KCl, 2 mm MgCl_2_, 40 μm GDP, 0.1% monothioglycerol, 0.5% Tween 20, 12% glycerol, 20 mm imidazole, 50 mm Tris-HCl (final pH 8.0) plus protease inhibitor mix (Complete EDTA-free; Roche) and 0.2 mg/ml lysozyme). Cells were ruptured at 4 °C by six 15-s pulses of sonic irradiation using a Branson cell disrupter (model W185D), separated by 15-s periods of cooling. The resulting lysate was clarified by centrifugation at 10,000 × *g* for 30 min at 4 °C. The clarified extract was subjected to IMAC on Ni^2+^-nitrilotriacetate-agarose beads (Qiagen) in high salt buffers (wash buffer: 300 mm KCl, 20 mm imidazole, 50 mm Tris-HCl (final pH 8.0); elution buffer: 300 mm KCl, 500 mm imidazole, 50 mm Tris-HCl (final pH 8.0)). Fractions containing the bulk of the purified septin complexes were combined, and the resulting pool (typically 5–6 ml) was passed over chitin-agarose beads (New England BioLabs) to remove three other endogenous *E. coli* gene products (ArnA, SlyD, and Can) that are other known contaminants in IMAC-based purifications (because, in *E. coli* NiCo21 (DE3), each of these gene products has been tagged with a chitin-binding domain). The septin complexes in the flow-through from the chitin-agarose were loaded using the 10-ml loop of an AKTA FPLC system (GE Healthcare) onto a Superdex 200 HiLoad 16/60 column (16 mm × 60 cm) (GE Healthcare) and subjected to size exclusion chromatography in septin buffer (300 mm KCl, 50 mm Tris-HCl (final pH 8.0)). Fractions were collected, and the proteins present in each were resolved by SDS-PAGE and visualized by staining with Coomassie Blue dye. The peak fractions containing the highest concentrations of stoichiometric septin complexes were pooled and used immediately for labeling by maleimide chemistry.

##### Expression and Purification of Bni5

Using the sequence- and ligation-independent cloning procedure, the yeast *BNI5* ORF was inserted in-frame with an N-terminal His_8_ tag followed by a human rhinovirus 3C protease cleavage site in the bacterial expression vector pH3c-LIC, in which transcription is driven by the phage T7 promoter and regulated in an isopropyl β-d-thiogalactopyranoside-dependent manner by a *lacO* element. This vector was introduced into *E. coli* NiCo21 (DE3), and the cells were induced and lysed, and the Bni5 protein was purified by IMAC, chitin-agarose, and size exclusion chromatography, essentially as described above for the bacterially expressed septin complexes.

##### Maleimide Labeling

The protein concentration of the purified septin complexes (or Bni5) was determined using the bicinchoninic acid method (Pierce BCA protein assay kit; Life Technologies, Inc.) (53). Samples (typically, 2–4 μg) were incubated with a 10-fold molar excess of reducing agent *tris*-(2-carboxyethyl)phosphine for 10 min at room temperature, desalted by passage through Sephadex G25 (8.3 ml; PD-10 column; GE Healthcare) in labeling buffer (300 mm KCl, 50 mm Tris (final pH 7.0)) to remove the *tris*-(2-carboxyethyl)phosphine, and labeled overnight at 4 °C with a 5-fold molar excess of the desired maleimide dye (AF488, AF555, and AF647; Life Technologies, Inc.). Excess dye was reacted with a 10-fold molar excess of DTT at room temperature for 10 min and removed by recapturing the (His)_6_-Cdc12-containing septin complex by IMAC chromatography on a HisTrap HP column (GE Healthcare) (wash buffer: 300 mm KCl, 20 mm imidazole, 50 mm Tris-HCl (final pH 8.0); elution buffer: 300 mm KCl, 500 mm imidazole, 50 mm Tris-HCl (final pH 8.0)). The dye-labeled protein was dialyzed overnight against septin buffer (300 mm KCl, 50 mm Tris-HCl (final pH 8.0)) in a Slide-A-Lyzer dialysis cassette (Life Technologies, Inc.) with a 10-kDa molecular weight cut-off. The BCA assay (corrected for the contribution of the dye) and measurement of fluorescence using a P-330 Nanophotometer (Implen) were used to determine the molar concentration of protein and dye, respectively, in the final sample. In these complexes, in which only a single protomer type, *e.g.* Cdc11, contains the sole Cys present, the efficiency of labeling was 0.7–0.8 dye molecules per this subunit. Labeling of only the proper protomer was verified by resolving the subunits by SDS-PAGE and analyzing them by imaging with a Typhoon Trio Variable Mode Imager equipped for fluorescence (GE Healthcare) to detect the dye and by staining with Coomassie Blue dye and examining with an Odyssey scanner (Licor Biosciences) to detect the protein. Using the same criteria, the labeling efficiency for Bni5, which contains three native cysteines, was 2.7 dye molecules per protein.

##### Spectroscopy and Data Analysis

Absorbance spectra of 25 nm donor dye (AF555)-labeled septin hetero-octamers alone, serial dilutions of 100 nm acceptor dye (AF647)-labeled septin hetero-octamers alone (unless otherwise specified), and mixtures of the two were measured, in triplicate, at room temperature after equilibration for 1 h in a cuvette (3-mm path length, 270-μl maximum volume) using a Cary Eclipse fluorescence spectrophotometer (Agilent). Unless specified otherwise, the final buffer conditions were 45 mm KCl, 50 mm Tris-HCl (pH 8.0). FRET values were obtained by subtracting the buffer-only background and correcting the emission of the acceptor at 671 nm for the contributions of both the donor and acceptor excited at 555 nm. The values reported represent the averages (±S.E.) of the triplicate samples. PCA (54) and subsequent data fitting was done using Matlab (The Mathworks) and its toolboxes for curve fitting and statistics. Alternately, semiquantitative sensitized emission techniques were used to obtain corrected FRET values, as previously described (55–57).

##### Fluorescence Microscopy

Microscope slides (3 inch × 1 inch × 1 mm) and coverslips (#1.5, 22 mm × 22 mm) were bathed in 2 m HCl for 30 min, rinsed five times in double distilled H_2_O (15 min per wash), rinsed once in ethanol, and air-dried. To coat the coverslip, 300 μl of 1 mg/ml poly-l-lysine was pipetted onto its surface, and after incubation for 30–60 min, it was rinsed twice with double distilled H_2_O, and air-dried. Purified septin complexes labeled with AF488 and AF555, respectively, were mixed and diluted to final concentration of 20 nm in 45 mm KCl, 50 mm Tris-HCl (pH 8.0) and incubated for 1 h at room temperature while protected from light. Samples (30 μl) of the resulting septin filament-containing solution were pipetted onto the coverslip and followed immediately by 200 μl of the same buffer to help disperse the protein more uniformly. The filaments were allowed to adhere to the poly-l-lysine surface for 30 min and then mounted on slides for imaging. The samples were viewed at room temperature using an Olympus IX81 microscope equipped with a 100X PlanApo Oil objective (numerical aperture 1.4, ∞/0.17) and an ORCA-ER CCD camera (Hamamatsu Photonics) (64.5 nm effective pixel size) controlled by MetaMorph (Molecular Devices). For AF488-labeled septins, an HQ 480/40 nm excitation filter and Q 505 long pass and HQ 535/50 nm emission filters were used (Chroma Technology Corp.). For AF555-labeled septins, an HQ 565-nm long pass and HQ 620/60-nm emission filters (Chroma Technology Corp.) were used. Unless otherwise specified, in all micrographs the *scale bar* represents 10 μm.

## Results

### 

#### 

##### End to End Assembly of Donor- and Acceptor-labeled Cdc11-capped Septin Hetero-octamers

Cdc11 is known to be the terminal subunit at each end of yeast septin hetero-octamers (22), which we have modeled on the basis of available crystal structures of mammalian septin complexes (37, 39, 40) using Phyre2 (58) (Fig. 1*A*). Prior work (18, 22, 59) has demonstrated that, in low salt buffer, native septin hetero-octamers assemble end on end via formation of an NC interface between Cdc11 subunits at the ends of adjacent rods to form a long filament (Fig. 1*B*). During their assembly, such individual filaments pair laterally in a highly cooperative manner. This arrangement seemed highly favorable for assessing the Cdc11-Cdc11 interaction via FRET because when donor dye-labeled Cdc11-capped rods are mixed with an equimolar amount of acceptor dye-labeled Cdc11-capped rods, on average, 50% of the resulting Cdc11-Cdc11 junctions should represent donor-acceptor pairs (Fig. 1*B*).

We selected as the donor-acceptor dye pair AF555 and AF647, two well characterized fluorophores, quite similar to Cy3 and Cy5, a well known FRET pair (60). As required for efficient FRET, the emission of the donor (AF555) shows good spectral overlap with the absorbance of the acceptor (AF647), with only a very modest tailing into the emission of the acceptor (Fig. 1*C*). Moreover, according to the manufacturer (Life Technologies, Inc.), the distance for 50% energy transfer (*R*_0_) between these two dyes is 5.1 nm, and, based on our modeling, at the Cdc11-Cdc11 NC interface, dye attached to E294C in the CTE of one Cdc11 protomer should approach the dye attached to E294C in the CTE of the other Cdc11 protomer at a distance of ∼3 nm.

To test these predictions, a preparation of otherwise Cys-less septin complexes containing Cdc11(E294C) was labeled with AF555 (Fig. 1*D*, *upper panels*), and another sample of the same complexes was labeled with AF647 (Fig. 1*D*, *lower panels*). Indeed, when increasing amounts of the acceptor dye-labeled rods were mixed with a fixed concentration of donor-labeled rods and excited only at the absorbance of the donor, the characteristic spectral features of FRET were clearly observed: a diminution of the donor emission with a concomitant and corresponding increase in emission from the acceptor (Fig. 1*E*), as also confirmed by PCA analysis (Fig. 1*E*, *inset*). The fact that only one principal component could be detected in the FRET spectrum (unless otherwise noted, this principal component accounted for >98% of the variability in our system) indicates that the FRET observed arises from the predicted bimolecular interaction of donor dye-labeled Cdc11 and acceptor dye-labeled Cdc11 at the Cdc11-Cdc11 junctions between rods and not from dye-dye interactions across the individual strands in paired filaments or other possible modalities, which would be expected to generate additional principal components.

**FIGURE 2.**
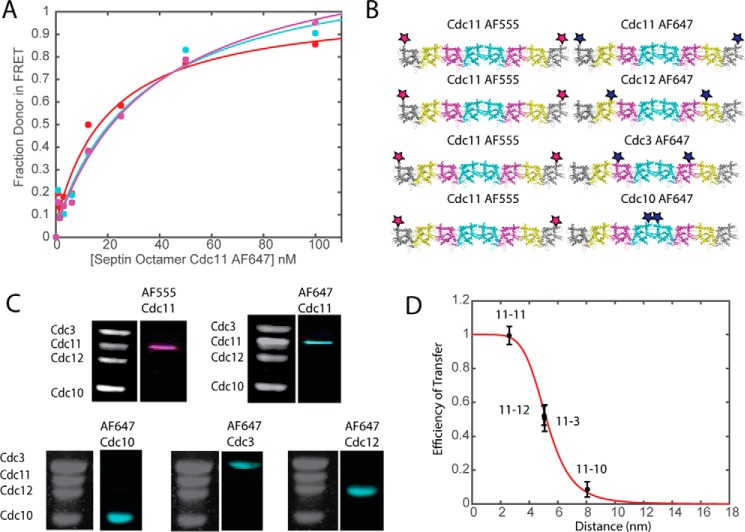
**FRET is sensitive to subunit labeling.** *A*, binding curves for Cdc11 AF555 and Cdc11 AF647 using varying concentrations of Cdc11^AF555^ (25 nm Cdc11^AF555^ (*red*), 35 nm Cdc11^AF555^ (*cyan*), 50 nm Cdc11^AF555^ (*magenta*)). *B*, schematic showing labeling of septin octamer at different subunits. The Cdc11^AF555^ is kept constant, whereas the acceptor (AF647) is transferred along the septin octamer. This schematic corresponds to the results in *C* and *D. C*, Coomassie gels and typhoon scans of labeled septin octamers with AF555 and AF647. *D*, the *red curve* shows the predicted efficiency of transfer from the FRET equation. The *black circles* with S.E. *error bars* correspond to experimental data for a 1:4 mixture of Cdc11^AF555^:acceptor-labeled septin. The data points are labeled for the labeled septins (donor-acceptor) in the FRET system.

Aside from revealing FRET arising from the expected close approach of donor-labeled Cdc11 and acceptor-labeled Cdc11 at the ends of the hetero-octamers, such titration experiments yield binding curves when replotted as the maximal corrected FRET observed at each of the known concentrations of acceptor dye-labeled rods that were added to the fixed known concentration of donor dye-labeled rods (Fig. 1*F*). Indeed, the expected approach to saturation was observed because the probability of formation of a FRET-competent Cdc11-Cdc11 junction between a donor dye-labeled rod and an acceptor dye-labeled rod should increase as the ratio of acceptor dye-labeled rods to donor dye-labeled rods increases. In addition, this very reproducible behavior indicates, contrary to indirect experiments suggesting the opposite (61), that, once assembled, a hetero-octamer is remarkably stable, *i.e.* subunits in different rods do not dissociate, intermix, and reassemble, at least over the time scale of our experiments. Moreover, assuming stochastic polymerization of donor- and acceptor-labeled rods, we could estimate the fraction of donor dye-labeled Cdc11-capped rods in end to end association with acceptor dye-labeled Cdc11-capped rods as the ratio of the concentration of the acceptor complexes divided by the total septin present (donor and acceptor complexes). In this way, such saturation curves (Fig. 1*F*) yield apparent *K_d_* values for the Cdc11-Cdc11 NC interface. The average *K_d_*^app^ value for the Cdc11-Cdc11 interaction derived from numerous experiments of this sort (Figs. 1*F* and 2*A*) was 25–30 nm ± 11 nm (range) with an apparent 1:1 stoichiometry (Table 1). Furthermore, assuming an “ideal” model, namely one Cdc11 binding to one Cdc11 (*AB*_max_ = 1) and a *K_d_* = 25 nm for that interaction, generated a binding isotherm nearly identical to the observed saturation curve (Fig. 1*F*), fully consistent with the FRET arising purely from end to end assembly of donor- and acceptor-labeled Cdc11-capped septin hetero-octamers.

**TABLE 1:** **Parameters from analysis of septin assembly with varying concentrations** Each value in the table represents the average of measurements made in triplicate and the ± values show the confidence interval calculated for *p* = 0.05.

FRET pair (donor-acceptor)	Donor concentration	Apparent *K_d_*	Apparent *AB*_max_ (fraction donor in FRET)
	*nm*	*nm*	
Cdc11^AF555^-Cdc11^AF647^	25	20.19 ± 18.97	0.97 ± 0.26
Cdc11^AF555^-Cdc11^AF647^	35	34.52 ± 34.87	1.20 ± 0.43
Cdc11^AF555^-Cdc11^AF647^	50	38.77 ± 27.72	1.28 ± 0.34
Shs1^AF555^-Cdc11^AF647^	25	10.78 ± 8.08	0.79 ± 0.15
Shs1^AF555^-Cdc11^AF647^	35	21.26 ± 28.72	0.90 ± 0.36
Shs1^AF555^-Cdc11^AF647^	50	27.51 ± 21.25	1.09 ± 0.27

##### Linear Organization of Septin Hetero-octamers

FRET can be used not only as a read-out for close association of a donor- and an acceptor-labeled molecule, but can also serve as a “molecular ruler” because the FRET efficiency (*E*) is strongly dependent on the distance between the donor and the acceptor (*r*), as well as on the 50% transfer distance (*R*_0_) for the dye pair used, where *F*_DA_ is the fluorescence intensity of the donor with the acceptor present, and *F*_D_ is the fluorescence intensity of the donor alone (Equation 1) (62).

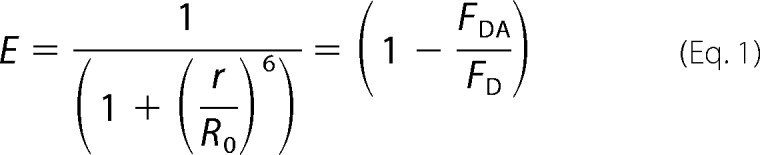


Given the fixed order of subunits in yeast septin hetero-octamers demonstrated both *in vitro* (22) and *in vivo* (63), the observed FRET between any given donor dye-labeled septin subunit and another acceptor-dye labeled protomer should be reproducibly sensitive to its position within the rod (Fig. 2*B*).

Again, to test the reliability of our system to exhibit this expected behavior, a preparation of otherwise Cys-less hetero-octamers containing Cdc11(E294C) labeled with the donor AF555 was titrated with preparations of otherwise Cys-less hetero-octamers containing, respectively, Cdc11(E294C), Cdc12(L310C), Cdc3(S407C), or Cdc10(R298C) that were labeled with the acceptor AF647 (Fig. 2*C*). As anticipated, we observed that FRET efficiency between Cdc11-Cdc11 donor-acceptor pairs was higher than for Cdc11-Cdc12, Cdc11-Cdc3, and Cdc11-Cdc10 donor acceptor pairs. The observed FRET efficiencies were used to calculate experimentally derived distances and plotted against those values (Fig. 2*D*). Although no crystal structure yet exists for *S. cerevisiae* septin complexes, our homology model (Fig. 1*A*) and the known diameter (4 nm) of the globular GTP-binding domain of yeast septin subunits estimated from EM (22) allowed us to predict, based on end to end assembly, the approximate distance between the positions of the fluorophores in the different septin pairs: Cdc11-Cdc11, ∼2.6 nm; Cdc11-Cdc12, ∼8.3 nm; Cdc11-Cdc3, ∼10.8 nm; and Cdc10, ∼16.7 nm. The experimentally derived values certainly follow the same trend as these predicted distances.

Especially noteworthy in this regard, however, was our finding that the calculated Cdc11-Cdc12 (5.02 nm) and Cdc11-Cdc3 (5.08 nm) distances were very similar. In the labeled Cdc12 and Cdc3 hetero-octamers, the acceptor dye is located near the base of the predicted α6 helix of the CTEs of these subunits, and ample evidence both *in vitro* and *in vivo* indicates that the CTEs of Cdc3 and Cdc12 are entwined in a coiled-coil interaction (22, 42, 59, 64). Given the position at which these acceptor dyes were installed, the fact that the measured distance between the Cdc11-Cdc12 and Cdc11-Cdc3 fluorophores was so similar suggests that, relative to the donor dye in Cdc11, the acceptor dyes in Cdc12 and Cdc3 are located in very close proximity to each other. This observation is consistent with and provides additional physical evidence for the presence of an intimate coiled-coil interaction between the CTEs of Cdc12 and Cdc3. As expected, there was a marked decrease in FRET between the terminal subunit (Cdc11) and the protomer (Cdc10) situated at the greatest distance away at the center of the rod (Fig. 2*D*).

Although the observed FRET values displayed a clear distance sensitivity, the experimentally calculated lengths deviated significantly from those predicted from our modeling. However, the “expected” distances were based on modeling against a rather low resolution (∼4 Å) crystal structure of mammalian septins (39). Also, our measurements were made using pairs of donor and acceptor fluorophores located at the very end of the α6 helix, which terminates the GTP-binding domain, at its junction with the long flexible CTE; thus, this position may have a significantly greater range of mobility than predicted. Finally, *in vivo*, septin rods assemble into filament-like structures on the cytosolic surface of the plasma membrane; however, when the septin rods assemble into filament-like structures in solution, they have a tendency to bundle laterally upon prolonged incubation or at a sufficiently high concentration (22, 44, 59). In principle, this bundling could contribute to the differences seen between the “expected” distances predicted by our modeling and the length values derived from our FRET measurements, if, for example, some filaments in such bundles were staggered (*i.e.* out of perfect register). Our fluorescence micrographs indicate that bundling occurs under our polymerization conditions (see supplemental Figs. S1–S4).

##### Effect of Ionic Strength on the Cdc11-Cdc11 NC Interface

**FIGURE 3.**
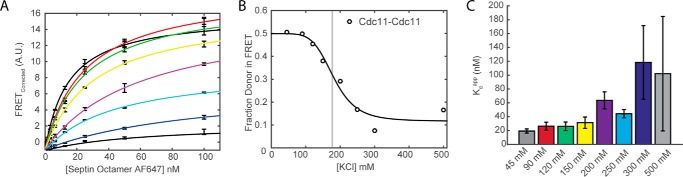
**Effect of ionic strength on septin assembly.** *A*, a series of binding curves for Cdc11-labeled septin subunits assembled under varying ionic strength using the FRET corrected method. *B*, using PCA and 1:1 mixture of Cdc11^AF555^:Cdc11^AF647^, apparent Hill coefficients and EC_50_ can be determined as a function of ionic strength. The *light gray line* illustrates the location of the EC_50_ value. *C*, bar graphs illustrate the values of binding constants corresponding to the *color-matched curves* in *A*.

There is ample evidence that septin filaments disassemble at high ionic strength into their constituent hetero-octamers (18, 20, 21) and, conversely, that polymerization of Cdc11-capped hetero-octamers into long paired filaments is favored at low ionic strength (22, 59). Thus, if our FRET system is reliably reporting filament assembly, the observed energy transfer between our donor dye-labeled Cdc11-capped rods and acceptor dye-labeled Cdc11-capped rods should be greatly diminished when the ionic strength is raised above the threshold required to disrupt the Cdc11-Cdc11 interaction. Indeed, when our standard titrations of donor-labeled rods with acceptor-labeled rods were conducted in buffers of increasing salt (KCl) concentration (Fig. 3*A*), the maximum observed FRET was progressively diminished, displaying a rather sharp transition with an EC_50_ of ∼180 mm KCl (Fig. 3*B*), exhibiting an apparent Hill coefficient above 5 (Table 2) and raising the *K_d_*^app^ from 20 to 30 nm to a value so weak and irreproducible (≫100 nm) that it was not possible to measure accurately (Fig. 3*C*).

**TABLE 2:** **Parameters from the analysis of septin assembly as a function of ionic strength** Each value in the table represents the average of measurements made in triplicate and the ± values show the confidence interval calculated for *p* = 0.05. Equimolar quantities of donor and acceptor were present while varying ionic strength.

FRET pair (donor-acceptor)	Ionic strength at EC_50_	Apparent Hill coefficient
	*mm*	
Cdc11^AF555^-Cdc11^AF647^	181.20 ± 46.50	5.50 ± 6.25
Shs1^AF555^-Cdc11^AF647^	197.90 ± 40.40	2.68 ± 1.39

##### Novel Insights about the Behavior of Shs1-capped Hetero-octamers

**FIGURE 4.**
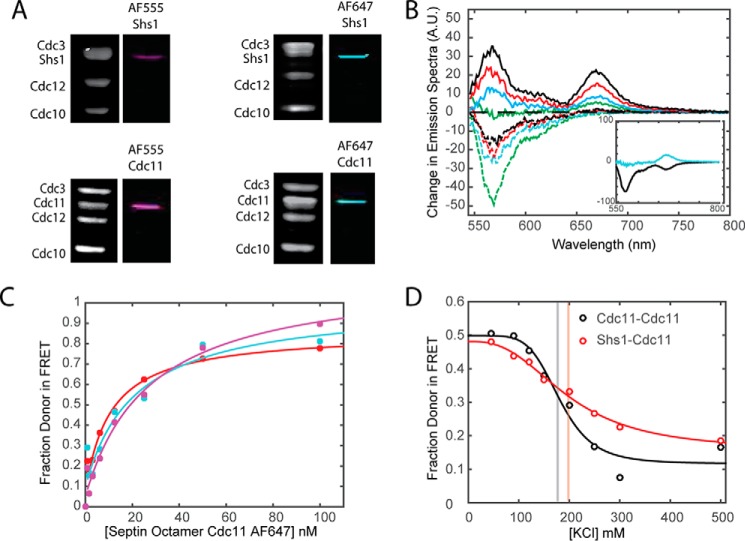
**Labeling of alternate terminal septin subunit (Shs1) does not establish a FRET system without Cdc11.** *A*, Coomassie gels and typhoon scans of labeled septin octamers with AF555 and AF647 using end subunits of Shs1 and Cdc11. *B*, FRET emission spectra with 25 nm Shs1^AF555^-labeled octamers recorded at increasing concentrations of Shs1^AF647^-labeled octamers: 0.78 nm (*dashed green*), 1.56 nm (*dashed cyan*), 3.13 nm (*dashed red*), 6.25 nm (*dashed black*), 12.5 nm (*solid green*), 25 nm (*solid cyan*), 50 nm (*solid red*), and 100 nm (*solid black*). The *inset* illustrates the result of the PCA with at least two principal components. *C*, titration curves of Cdc11^AF647^ capped octamers with Shs1^AF555^ capped octamers at varying concentration of Shs1^AF555^ (25 nm Shs1^AF555^ (*red*), 35 nm Shs1^AF555^ (*cyan*), and 50 nm Shs1^AF555^ (*magenta*)). *D*, using PCA, Hill coefficients and IC_50_ can be determined as a function of ionic strength. The *light red line* illustrates the location of the IC_50_ value. The Cdc11^AF555^-Cdc11^AF647^ (*black line*) is included for reference.

Aside from Cdc3, Cdc10, Cdc11, and Cdc12, mitotically growing yeast cells express a fifth septin, Shs1 (551 residues), that is most closely related to but 33% larger than Cdc11 (415 residues). Prior work has demonstrated that Shs1 can replace Cdc11 and thus serve as an alternative terminal subunit in yeast septin hetero-octamers (25). Indeed, like Cdc11-capped hetero-octamers (Fig. 4*A*, *lower panels*), we were able to prepare stable, otherwise Cys-less septin complexes containing Shs1(E344C) that we could efficiently label with either the donor AF555 or acceptor AF647 dyes (Fig. 4*A*, *upper panels*). These reagents allowed us to address several previously unresolved questions about the properties of such Shs1-capped rods.

First, EM imaging indicates that, in low salt buffers, Shs1-capped hetero-octamers bundle and form rather thick arcs, rings, spirals, and bird's nest-like arrangements (25). Given the density of these structures, it has not been possible to discern definitively whether or not Shs1-capped hetero-octamers have any capacity to join end on end via an Shs1-Shs1 NC interface. To address this question, a fixed concentration of donor dye-labeled Shs1(E344C)-containing rods was titrated with increasing concentrations of acceptor dye-labeled Shs1(E344C)-containing rods. Unlike the robust FRET observed for the such donor-acceptor titrations with Cdc11-capped rods (Fig. 1*E*), no detectable FRET occurred in the Shs1-Shs1 donor-acceptor mixtures, as evidenced by the fact that there was no internally consistent decrease in the emission of the donor and no corresponding enhancement in the emission of the acceptor above the background contribution of its increasing concentration (Fig. 4*B*). Thus, as judged by this criterion, Shs1-capped hetero-octamers are unable to engage in any persistent end on end association. Moreover, PCA of the spectral data showed two distinct principal components (Fig. 4*B*, *inset*), suggesting that the spectra recorded arise from at least two classes of weak fluorophore interactions, consistent with the complex structures formed by Shs1-capped hetero-octamers on EM grids (25). Indeed, even at the macroscopic level of the fluorescence microscope, fluorescently tagged Shs1-capped complexes formed curved structures and even some rings (supplemental Fig. S1, *left panel*).

Second, it has been shown biochemically that purified Shs1 can associate with purified Cdc11 *in vitro* (20) and, as viewed in the EM, that titering a solution of Shs1-capped hetero-octamers with increasing amounts of Cdc11-capped hetero-octamers widens the diameter and thins out the edges of the rings formed from Shs1-capped complexes, suggesting that Cdc11-capped rods can intercalate in some fashion into structures composed of Shs1-capped rods and alter their supramolecular organization (25). These findings suggest that Cdc11-capped complexes and Shs1-capped complexes might be able to form heterotypic NC junctions. Consistent with this conclusion, we recently provided genetic evidence that such heterotypic NC junctions likely occur in the cell (65). However, there previously has been no direct and incisive means to confirm that Shs1-capped hetero-octamers are able to form heterotypic NC junctions with Cdc11-capped hetero-octamers. To test this possibility directly, we titrated several fixed concentrations of donor dye-labeled Shs1-capped rods with increasing concentrations of acceptor dye-labeled Cdc11-capped rods. In marked contrast to the Shs1-Shs1 donor-acceptor mixtures, the Shs1 donor-Cdc11 acceptor mixtures exhibited robust FRET (Fig. 4*C*), equivalent to that displayed by Cdc11-Cdc11 donor-acceptor mixtures (Figs. 1*F* and 2*A*). The average *K_d_*^app^ value for the Shs1-Cdc11 interaction derived from these binding curves was ∼20 nm ± 10 nm (range) (Table 1), a slightly higher affinity than measured for the Cdc11-Cdc11 NC interaction. Likewise, the effect of increasing salt concentration on the stability of the Shs1-Cdc11 association displayed a somewhat broader transition and a somewhat higher EC_50_ of ∼200 mm KCl than for the Cdc11-Cdc11 interaction (Table 2), again consistent with the Shs1-Cdc11 interaction being somewhat tighter than the Cdc11-Cdc11 interaction. Thus, we conclude that Shs1-capped hetero-octamers are indeed able to engage in a heterotypic end on end interaction with Cdc11-capped hetero-octamers.

Interestingly, at the macroscopic level, in 1:1 mixtures of fluorescently tagged Shs1-capped septin complexes and Cdc11-capped septin complexes, the two dyes are clearly incorporated into the same structures (supplemental Figs. S2*A* and S4 *A*); however, the Shs1-containing regions are discontinuous and appear embedded within a more continuous matrix of Cdc11-containing regions (supplemental Figs. S2*A* and S4*A*, *white arrowheads*). This organization could reflect the demonstrated capacity of Shs1-capped hetero-octamers for lateral bundling (25), such that even if an Shs1-capped rod is incorporated into these structures via its end to end association with Cdc11-capped rods, it could still nucleate lateral binding of additional Shs1-capped rods at the same location, resulting in the observed puncta. Presumably, the properties of such mixed assemblies give septin-based structures the flexibility and plasticity necessary to form the lattice- and gauze-like arrangements, and other supramolecular architectures, that have been observed in cells by freeze-fracture (66), cryo-electron tomography (23), and other EM methods (24).

##### Analysis of the Role of Conserved Structural Elements in Septin-Septin Interaction

**FIGURE 5.**
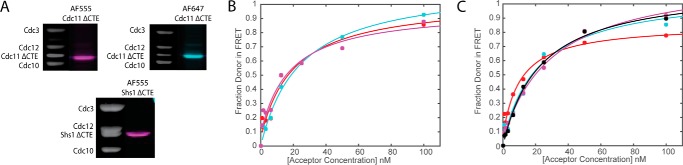
**Effect of deletion of the terminal subunit C-terminal extension on septin octamer assembly by FRET.** *A*, Coomassie gels and typhoon scans of labeled septin octamers with AF555 and AF647 using end subunits of Shs1 ΔCTE and Cdc11 ΔCTE. *B*, binding curves for Cdc11^AF555^-Cdc11^AF647^ (*red*), Cdc11^AF555^-Cdc11 ΔCTE^AF647^ (*cyan*), and Cdc11 ΔCTE^AF555^-Cdc11 ΔCTE^AF647^ (*magenta*). *C*, binding curves for Shs1^AF555^-Cdc11^AF647^ (*red*), Shs1^AF555^-Cdc11 ΔCTE^AF647^ (*cyan*), Shs1 ΔCTE^AF555^-Cdc11^AF647^ (*magenta*), and Shs1 ΔCTE^AF555^-Cdc11 ΔCTE^AF647^ (*black*). For *B* and *C*, *dots* represent measured data, and *color-coded lines* represent the corresponding binding curve fit.

In the original x-ray structure of the human Sept7-Sept6-Sept2-Sept2-Sept6-Sept7 hetero-hexamer, each subunit was truncated to remove its CTE to promote crystallization (39), so no information about the disposition of those structural elements (Fig. 1*A*, *arrows*) was provided. Subsequently, the isolated CTEs of these same septins have been studied as purified recombinant proteins in solution, and it has been reported that the CTEs of Sept6 and Sept7 (equivalent to yeast Cdc3 and Cdc12, respectively) are able to form an elongated heterodimer with high α-helical content (presumably, a coiled coil) with a *K_d_* of 15.8 nm (67). This value is in the same range as the Cdc11-Cdc11 and Shs1-Cdc11 interactions shown here. Moreover, although cells in which either *cdc11*(Δ*CTE*) or *shs1*(Δ*CTE*) mutants are expressed as sole source of these septins are viable, their growth and morphology are not normal (20, 64). Hence, we sought to assess the contributions of the corresponding CTEs of Cdc11 and Shs1 to Cdc11-Cdc11 homotypic and Shs1-Cdc11 heterotypic association. Cdc11 and Shs1 contain CTEs that are similar, apart from a 45-residue insert (residues 401–446) found in Shs1. Removal of the CTE (residues 306–415) from Cdc11 or of the CTE (residues 349–551) from Shs1 did not affect the engineered Cys in α6 (E294C in Cdc11 and E344C in Shs1, respectively) that we used for maleimide labeling and do not affect the formation of stable hetero-octameric complexes (25, 50). Hence, we prepared, labeled and analyzed otherwise Cys-less complexes containing either Cdc11(E294C ΔCTE) or Shs1(E344C ΔCTE) (Fig. 5*A*). We found that absence of the CTE from Cdc11 in the acceptor dye-labeled rods or from both the donor dye-labeled rods and the acceptor dye-labeled rods did not have a significant effect on the FRET observed (Fig. 5*B*) or on the *K_d_*^app^ value for the Cdc11-Cdc11 interaction derived from such binding curves (Table 3). Likewise, we found that removal of the CTE from Shs1 or from Cdc11 or from both did not have a significant effect on the FRET observed (Fig. 5*C*) or on the *K_d_*^app^ value for the Shs11-Cdc11 interaction derived from such binding curves (Table 3). Likewise, at the macroscopic level, fluorescently tagged hetero-octamers capped with Cdc11(ΔCTE) formed polymerized structures with either Cdc11-capped hetero-octamers or Cdc11(ΔCTE)-capped hetero-octamers with equal efficiency (supplemental Fig. S3, *B* and *C*). Similarly, the presence or absence of the CTE on Shs1 or Cdc11, or both, did not prevent the formation of colocalized polymerized structures closely resembling those formed between intact Shs1-capped and intact Cdc11-capped septin complexes (supplemental Fig. S2, *B–D*). These results are consistent with our recent genetic evidence that, unlike heterodimeric coiled coil formation by the CTEs of Cdc3 and Cdc12 that contributes to the strength of their interaction (20, 22, 59), the CTEs of Cdc11 and Shs1 do not make a major contribution to either Cdc11-Cdc11 or Shs1-Cdc11 interaction but rather contribute to the efficiency of recruitment *in vivo* of at least one septin-associated protein to the septin-containing structures at the bud neck (65).

**TABLE 3:** **Parameters for the analysis of septin assembly with ΔCTE septins** Each value in the table represents the average of measurements made in triplicate and the ± values show the confidence interval calculated for *p* = 0.05. The donor was held at 25 nm, and FRET was measured in the absence and presence of increasing concentrations of acceptor up to 100 nm.

FRET pair (donor-acceptor)	Apparent *K_d_*	Apparent *AB*_max_ (fraction donor in FRET)
	*nm*	
Cdc11^AF555^-Cdc11 ΔCTE^AF647^	24.11 ± 11.45	1.15 ± 0.17
Cdc11 ΔCTE^AF555^-Cdc11 ΔCTE^AF647^	17.68 ± 17.65	0.88 ± 0.24
Shs1^AF555^-Cdc11 ΔCTE^AF647^	22.24 ± 16.45	1.06 ± 0.23
Shs1 ΔCTE^AF555^-Cdc11^AF647^	30.38 ± 12.83	1.17 ± 0.17
Shs1 ΔCTE^AF555^-Cdc11 ΔCTE^AF647^	22.83 ± 7.95	1.13 ± 0.12

Another helical element, α0, observed in the crystal structure of a human Sept2-Sept2 NC dimer (39), contributes some residues to the buried surface at this interface (39) and has been implicated previously in the strength of septin-septin interaction both *in vitro* (22) and *in vivo* (63). However, a tract of basic residues found within α0 also appears to be sufficiently surface-exposed so as to contribute to the association of septin complexes with phosphatidylinositol 4,5-bisphosphate in the plasma membrane (59, 64, 68). Sequence alignments and modeling indicate that all of the yeast mitotic septins have an α0. The apparent α0 in Cdc11 and Shs1 corresponds to residues 6–19 and 7–20, respectively. As analyzed by deposition onto carbon-coated grids and examination in the EM, septin hetero-octamers capped with Cdc11(Δ2–18), which removes essentially the entire α0 hence, termed Cdc11(Δα0), were unable to polymerize in solution into filaments (22). In marked contrast, hetero-octamers capped with Cdc11(Δα0) were able to assemble end on end to form filaments when polymerized on the surface of a PtdIns4, 5P_2_-containing lipid monolayer (59), indicating some significant residual capacity of the Cdc11(Δα0)-capped rods to associate via the Cdc11-Cdc11 NC interface.

**FIGURE 6.**
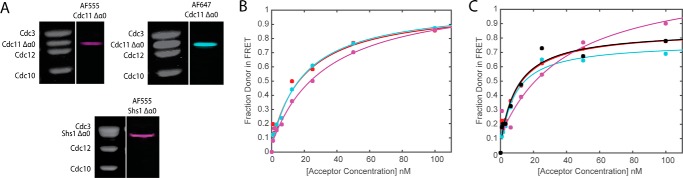
**Effect of deletion of the terminal subunit α0 helix on septin octamer assembly by FRET.** *A*, Coomassie gels and typhoon scans of labeled septin octamers with AF555 and AF647 using end subunits of Shs1 Δα0 and Cdc11 Δα0. *B*, binding curves for Cdc11^AF555^-Cdc11^AF647^ (*red*), Cdc11^AF555^-Cdc11 Δα0^AF647^ (*cyan*), and Cdc11 Δα0^AF555^-Cdc11 Δα0^AF647^ (*magenta*). *C*, binding curves for Shs1^AF555^-Cdc11^AF647^ (*red*), Shs1^AF555^-Cdc11 Δα0^AF647^ (*cyan*), Shs1 Δα0^AF555^-Cdc11^AF647^ (*magenta*), and Shs1 Δα0^AF555^-Cdc11 Δα0^AF647^ (*black*). For *B* and *C*, *dots* represent measured data, and *color-coded lines* represent the corresponding binding curve fit.

To explore this question further, we prepared, labeled, and analyzed otherwise Cys-less complexes containing either Cdc11(Δα0 E294C) or Shs1(Δ2–18/Δα0 E344C) (Fig. 6*A*). Titrating Cdc11-capped donor dye-labeled rods with Cdc11(Δα0) acceptor dye-labeled rods yielded FRET behavior and an apparent affinity for Cdc11-Cdc11 interaction that was indistinguishable from that observed with wild-type Cdc11 acceptor dye-labeled rods (Fig. 6*B* and Table 4); however, when α0 was absent from the Cdc11 in both the donor dye-labeled rods and the acceptor dye-labeled rods, there was a modest, but measurable, reduction in the apparent strength of the Cdc11-Cdc11 interaction (Fig. 6*B* and Table 4). Similarly, when mixtures of Shs1-capped complexes and Cdc11-capped complexes were examined, the most striking effect was observed upon removal of α0 from Shs1 in donor dye-labeled complexes, which weakened the *K_d_*^app^ value for the Shs1-Cdc11 interaction by 4–5-fold (Fig. 6*C* and Table 4); however, the noise in these particular measurements makes this value only very approximate. Nonetheless, when both Shs1 and Cdc11 lacked α0, interaction strength was improved (Fig. 6*C* and Table 4). PCA (not shown) of the same spectral data indicated maintenance of a single principal component in all these mutant combinations, supporting the view that 1:1 Cdc11-Cdc11 and Shs1-Cdc11 interactions were still able to occur *in vitro* even when these subunits lack their α0 element. Thus, it appears that those residues of α0 thought to be buried at the NC interface do not contribute in a major way to the binding energy of this interaction, at least in solution.

**TABLE 4:** **Parameters for the analysis of septin assembly with Δα0 septins** Each value in the table represents the average of measurements made in triplicate, and the ± values show the confidence interval calculated for *p* = 0.05. The donor was held at 25 nm, and FRET was measured in the absence and presence of increasing concentrations of acceptor up to 100 nm.

FRET pair (donor-acceptor)	Apparent *K_d_*	Apparent *AB*_max_ (fraction donor in FRET)
	*nm*	
Cdc11^AF555^-Cdc11 Δα0^AF647^	19.49 ± 8.23	1.00 ± 0.12
Cdc11 Δα0^AF555^-Cdc11 Δα0^AF647^	33.21 ± 19.17	1.07 ± 0.22
Shs1^AF555^-Cdc11 Δα0^AF647^	8.13 ± 4.47	0.75 ± 0.10
Shs1 Δα0^AF555^-Cdc11^AF647^	40.58 ± 58.31	1.28 ± 0.61
Shs1 Δα0^AF555^-Cdc11 Δα0^AF647^	9.89 ± 8.93	0.80 ± 0.18

However, our complementary analysis of the ability of these fluorophore-labeled complexes to assembly into higher order structures at the macroscopic level, as assessed by fluorescence microscopy, provided evidence that Cdc11 and Shs1 lacking their α0 are less stable and have a tendency to mis- or unfold, as judged by the fact that the filamentous networks formed are much shorter and often contained what look like aggregates, especially when only Cdc11(Δα0)-capped hetero-octamers were present (supplemental Fig. S3, *D* and *E*). Similarly, in the case of mixtures of Shs1-capped complexes with Cdc11-capped complexes, removal of α0 from either or both of these septins altered their ability to form the higher order structures observed with complexes capped with their wild-type counterparts. When Cdc11(Δα0) complexes were combined with Shs1 complexes, there was less colocalization of Cdc11(Δα0) with Shs1 and an increase in the formation of Shs1-containing puncta (supplemental Fig. S4*B*). Conversely, Shs1(Δα0) complexes combined with Cdc11 complexes exhibited increased colocalization, suggesting that removal of α0 from Shs1 permits its more facile integration into Cdc11-containing structures (supplemental Fig. S4*C*). When Shs1(Δα0) complexes were combined with Cdc11(Δα0) complexes, however, very few organized structures of any sort were formed from either septin-containing complex (supplemental Fig. S4*D*). These findings could be best explained by a role for α0 in maintaining the stable three-dimensional fold in both Cdc11 and Shs1.

##### Binding and Localization of a Septin-associated Protein

In addition to its use in monitoring assembly of fluorophore-labeled building blocks into a polymeric structure and its utility in gauging approximate distances among fluorophore-labeled constituents of multiprotein complexes, FRET can also be used to determine whether an exogenously added, heterologous protein can associate with a pre-existing complex and the binding affinity and preferred binding site of that interaction. To test the utility of our system for this purpose, we examined the association of four derivatives of Cdc11-capped septin complexes, each donor dye-labeled on one of its component subunits and assembled into filaments at low salt concentration, with acceptor dye-labeled Bni5.

*BNI5* was isolated as a dosage suppressor of *cdc12-6* and other temperature-sensitive septin mutants, and yeast two-hybrid analysis *in vivo* and GST pulldown assays *in vitro* indicated that Bni5 physically associates most strongly with Cdc11 (69). Subsequent work has demonstrated that Bni5 serves as an adaptor to link septin filaments at the bud neck to the yeast type II myosin (Myo1) necessary for contractile ring formation prior to cytokinesis (65, 70, 71).

**FIGURE 7.**
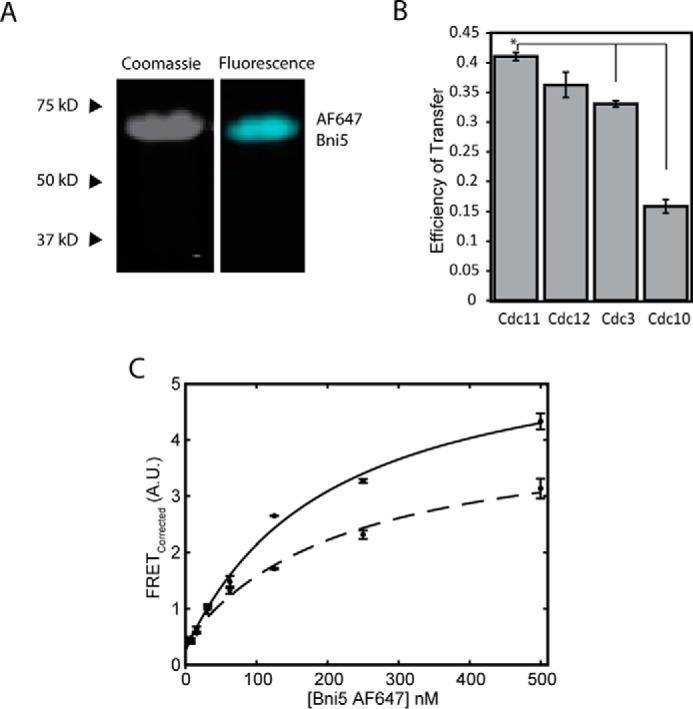
**Bni5 binding to septin hetero-octamers.** *A*, Coomassie gel and typhoon scan of Bni5 purified and labeled with AF647 including position of MW ladder. *B*, bar graph showing the measured efficiency of transfer for acceptor dye-labeled Bni5 with each donor dye-labeled septin subunit in Cdc11-capped hetero-octamers. Analysis of variance (*p* < 0.05) shows significant increase in efficiency of transfer between Bni5 with Cdc11 over Cdc3 and Cdc10-labeled subunits. *C*, binding curves of Bni5^AF647^ (maximum, 500 nm) with Cdc11^AF555^ (15 nm (*dashed line*) and 25 nm (*solid line*)) septin octamers with measured *K_d_* values of 221.5 ± 148.5 and 210.2 ± 99.9 nm, respectively (mean ± values show the confidence interval calculated for *p* = 0.05).

As an initial test, we labeled purified native Bni5, which contains three endogenous Cys (Cys-144, Cys-266, and Cys-375), with the acceptor dye (Fig. 7*A*). The efficiency with which we were able to label Bni5 with acceptor dye (2.7 AF647 molecules per protein) indicates that all three of Cys residues are solvent-exposed, consistent perhaps with structural predictions, suggesting that Bni5 is a very elongated, nearly all-α-helical protein (65). Because of the multiple dyes present, we anticipated that if FRET was observed between the acceptor-labeled Bni5 and the donor dye-labeled septin filaments, any values obtained from it for apparent affinity and distance estimates would only be approximate. Nonetheless, we found that acceptor dye-labeled Bni5 showed readily detectable FRET with septin filaments containing donor dye-labeled Cdc11 and progressively lower FRET with septin filaments where the donor dye was located on another subunit (Fig. 7*B*), in agreement with all of the prior evidence that Bni5 associates preferentially with Cdc11 in Cdc11-capped hetero-octamers. The *K_d_*^app^ derived for the Bni5-Cdc11 interaction from multiple titrations of donor dye-labeled Cdc11(E294C)-containing filaments was ∼200 nm (Fig. 7*C*). This apparent affinity is in remarkably good agreement with a value (∼300 nm) reported for binding of Bni5 to septin complexes measured by the technique of surface plasmon resonance (44).

## Discussion

### 

#### 

##### FRET Provides a Versatile Assay for Monitoring Septin Mechanics

Prior to this work, the only assay available to observe septin assembly *in vitro* was by visual inspection of septins tagged by fusion to large genetically encoded fluorogenic proteins under the fluorescence microscope (43). Although this technique is useful to observe the dynamics of formation of septin structures on a large scale, the protein-protein interactions intimately involved in the mechanism of septin assembly at the nanometer scale are inaccessible at the level of resolution of fluorescence microscopy. By contrast, our development of a FRET-based method enabled us to address detailed questions about the assembly mechanics and subunit interactions in septin complexes.

One major benefit of FRET is that the efficiency of the energy transfer between small, organic dye fluorophores is dependent on the distance between them in a predictable fashion and on scales relevant for septin biology. Thus, by placing FRET-compatible fluorophores on different combinations of subunits, we were able to provide independent confirmation that Cdc11-capped septin hetero-octamers assemble into filaments by end on end formation of homotypic Cdc11-Cdc11 junctions and not via any other sort of subunit-subunit interactions. Our measurements also provide the first quantitative estimate of the strength of the Cdc11-Cdc11 NC interface, which has a remarkably high affinity (*K_d_*^app^ = ∼30 nm). Moreover, we were able to demonstrate, conversely and for the first time, that Shs1-capped hetero-octamers are incapable of engaging in end on end association via homotypic Shs1-Shs1 interaction. Revealingly, however, and yet again for the first time, our FRET method showed that Shs1-capped hetero-octamers are capable of engaging in heterotypic Shs1-Cdc11 junctions, as had been inferred from purely circumstantial evidence based on genetic arguments in our prior studies (65). Currently, there is no evidence either *in vitro* (25) or *in vivo* (25, 63, 64, 73) that a heteroctamer can be heterotypically capped with a Cdc11 at one end and Shs1 at the other. Moreover, at a more macroscopic scale, we found that these Shs1-Cdc11 junctions are formed at a distinctly lower frequency than Cdc11-Cdc11 because Shs1-capped rods are incorporated within higher order Cdc11-containing structures in a highly dispersed and irregularly periodic manner. This behavior is in keeping with one of the likely physiologic roles for incorporation of Shs1-capped hetero-octamers into the filaments composed of Cdc11-capped hetero-octamers, namely to assist in imparting curvature to generate and control the diameter of the septin rings at the bud neck, whose size changes during progression through the yeast cell division cycle (24, 32). This hypothesis is supported by experiments in which changing the relative stoichiometry of Shs1- and Cdc11-capped hetero-octamers altered the diameter and thickness of rings observed under the EM (25) and by the fact that although *shs1*Δ mutants are viable, the efficiency of cytokinesis in such cells is clearly compromised (73, 74).

##### Conditions Influencing Septin Filament Assembly

A valuable feature of our FRET-based assay and another advantage over studies conducted using fluorescence microscopy is that it is readily accessible to manipulation of the solvent conditions. There are many parameters that could affect septin assembly that might also be regulated in the cell, such as the guanine nucleotide concentration or pH.

Here, however, we chose to examine the effect of ionic strength because of its well characterized influence on the state of septin filament assembly *in vitro* demonstrated in prior work (18, 22). Using our FRET system, we found that the ability of Cdc11-capped rods to polymerize end on end into filaments is indeed salt-sensitive. The transition between filaments and isolated rods was very sharp (an apparent Hill coefficient >5) and occurred at an EC_50_ for the molarity of KCl just slightly higher (∼180 mm) than that thought to represent the tonicity inside cells (equivalent to 120–150 mm) (75). This finding suggests that, at the ionic strength inside cells, the rod to filament equilibrium may be poised to be ultrasensitive to changes in other septin effectors (*e.g.* changes in the concentrations of particular septin subunits, GTP, and/or septin-associated proteins, or changes in post-translational modifications on septin or septin-associated proteins). If so, it would give cells the capacity to rapidly remodel septin-based structures in response to the appropriate cellular cues. Indeed, the septin cytoskeleton undergoes a variety of dramatic structural changes as the cell cycle proceeds: formation of a septin cap or nascent hoop at the incipient bud site in late G_1_; expansion/extension of the hoop into an hourglass-shaped collar in S to late anaphase; splitting or collapse of the collar into two bands at the onset of cytokinesis; and breakdown of all observable higher order septin structure prior to initiation of the next cell cycle (29, 30).

In further regard to the effect of ionic strength and given that previous observations in our own and in other laboratories have shown that septin hetero-octamers themselves are remarkably stable even at salt concentrations as high as 300–500 mm NaCl or KCl (18, 20–22), the salt-sensitive interaction responsible for salt-induced filament disassembly is clearly the Cdc11-Cdc11 NC interface. In this same regard, and given that our FRET approach was able to definitely show that Shs1-Cdc11 NC junctions are able to form robustly and have an apparent affinity (*K_d_*^app^ ∼20 nm) even greater than Cdc11-Cdc11 junctions, it is tempting to speculate that the reported extensive and cell cycle-dependent phosphorylation of Shs1 (76, 77), which does not appear to be required for Shs1 function *per se* (64), occurs as a means to enhance the efficiency of disassembly of the residual septin filaments and rings at the bud neck at the end of each cell cycle.

##### Analysis of Conserved Structural Elements in Septin Organization

In addition to examining the properties of wild-type septin complexes, we also used our FRET methodology to explore the effect of altering certain recognized structural elements in septin monomers on the assembly properties of hetero-octamers containing them. Here, in particular, we probed the roles of the CTE and α0 helix in Cdc11 and in Shs1 in the formation of both homotypic Cdc11-Cdc11 interaction and in heterotypic Shs1-Cdc11 association.

Deletion of the CTE, even when removed from Cdc11 in both the donor dye-labeled Cdc11-capped complexes and the acceptor dye-labeled Cdc11 capped complexes, did not prevent formation of homotypic Cdc11-Cdc11 NC junctions or affect their apparent affinity. Likewise, deletion of the CTE from either Shs1 or Cdc11, or both, did not significantly perturb formation of heterotypic the Shs1-Cdc11 NC junction. Thus, the CTEs of Cdc11 and Shs1 do not contribute to either the Cdc11-Cdc11 or the Shs1-Cdc11 interaction, consistent with recent genetic evidence that these CTEs serve instead to enhance the recruitment of at least one septin-associated protein (65).

Our FRET system also allowed us to interrogate and answer a lingering issue about another structural element, α0, thought to reside at the NC interface. However, there have been conflicting views about the degree to which α0 is buried or solvent-exposed and how much it contributes to the strength of the NC interaction (39, 63, 64). We found that removal of the α0 helix had no major effect on *K_d_*^app^ for either the Cdc11-Cdc11 or the Shs1-Cdc11 interaction. Indeed, as first shown using lipid monolayers on EM grids to capture septin filament polymerization (59), hetero-octamers capped with Cdc11(Δα0) retain the capacity to assemble into filaments, in contradiction to earlier work (22) in which similar preparations were examined for filament assembly in solution by then depositing the material on EM grids for visualizing by negative staining in the EM. Thus, our findings agree with the more recent data. It is possible that the original discrepancy arose because in the earlier study preformed filaments may have disassembled under the conditions required for processing of the samples, which involved solutions of heavy metals that have a high ionic strength.

Moreover, we found (see images in supplemental Figs. S1–S4) that upon assembly, hetero-octamers capped with Cdc11(Δα0) have a much greater tendency than hetero-octamers capped with wild-type Cdc11 to accumulate puncta and form larger order aggregates, and in the original study, such structures (because they did not resemble long paired filaments) may have been overlooked. In any event, our findings indicate that a lack of the α0 helix destabilizes Cdc11 and Shs1, resulting in a greater propensity for these subunits to undergo unfolding or misfolding.

Furthermore, in agreement with our FRET results indicating little direct role for the α0 helix in formation of the Cdc11-Cdc11 or Shs1-Cdc11 NC interfaces, there is ample evidence that the tract of basic residues in the α0 helix of mitotic septin subunits Cdc10 (59), Cdc11, and Shs1 (63, 64) is sufficiently solvent-exposed to contribute to the interaction of septin filaments with phosphatidylinositol 4,5-bisphosphate in the plasma membrane and is required for the biological function of these septins *in vivo*. It should be conceded that one drawback of our FRET method in solution, as it presently stands, is that it cannot assess interactions of septins with membranes or membrane surrogates.

##### Binding of a Septin-associated Protein

Finally, we demonstrated that we could apply our FRET approach to delineate the affinity of binding and the location of binding of a septin filament-associated protein. There are a plethora of gene products that, when GFP-tagged, localize to the yeast bud neck (29, 78), but in only a very few cases has it been determined which of these proteins physically associates directly with a septin or is localized at the bud neck indirectly by steric trapping or through association with the plasma membrane or with another protein that does bind directly to a septin. Our FRET method, because it uses purified components, can distinguish between these two extremes for any bud neck-localized protein that can be expressed and purified as a recombinant protein or purified to homogeneity from yeast cells.

In this regard and as a test case, we examined the association of Bni5 (Bud neck-interacting protein 5) (69). We found, using our FRET assay, that Bni5 is indeed capable of binding directly to septin filaments and shows a strong preference for association with Cdc11, in agreement with results obtained by others using different *in vivo* and *in vitro* methods. Moreover, our analysis yielded a *K_d_*^app^ value for the Bni5-Cdc11 interaction (∼200 nm) quite similar to that (∼300 nm) obtained by a completely different and independent biophysical method conducted by others (44). Preparing single-Cys derivatives of Bni5 will allow refinement of our binding curves to obtain more accurate assessment of its affinity and, especially, its orientation at its binding site. Furthermore, our FRET approach can be extended to defining by mutagenesis the residues in both Bni5 and Cdc11 that are essential for their high affinity interaction.

Clearly, our system can be extended to interrogate any septin-interacting protein to determine the affinity of its binding, to pinpoint which septin subunit constitutes its preferred binding partner, and to determine its orientation relative to other landmarks in the hetero-octamer, such as the edge from which all the CTEs project or the opposite edge (Fig. 1*A*). Indeed, in this regard, we have available functional single-Cys substitutions situated at various positions in and around the surface of each of the mitotic subunits (50) that could be used systematically to acquire information about such distance constraints. Similarly, with proper stopped flow instrumentation, our FRET assay could be modified to obtain kinetic parameters for polymerization of septin hetero-octamers into filaments and to determine the rate of association of septin-binding proteins with filaments. Finally, with the proof of principle we have provided here, our FRET-based techniques could also be extended to analyze the much more complex interplay among the 13 different human septin subunits and their multifarious splice variants and other isoforms (5, 79) and to address the organization and polymerization behavior of the complexes comprising them. Past work with other biopolymers, such as actin (72), has shown that the combination of spectroscopy with microscopy is exceedingly powerful for determining structure-function relationships. Relative to other cytoskeletal biopolymers, techniques for studying septins have been more rudimentary. We hope that the establishment of our FRET-based assay will provide a platform for greater understanding of how septins, their binding partners, and their regulatory factors are linked to the biological functions of these fascinating proteins.

## Author Contributions

E. A. B. and J. T. designed the research plan; E. A. B., E. W. V., and D. D. devised the experimental procedures; E. A. B. and E. W. V. conducted the experiments and analyzed the data; and E. A. B. and J. T. wrote the manuscript.

## Supplementary Material

Supplemental Data

## Footnotes

5 The abbreviations used are:

CTE: C-terminal extension
AF488: Alexa Fluor 488 maleimide
AF555: Alexa Fluor 555 maleimide
AF647: Alexa Fluor 647 maleimide
IMAC: immobilized metal ion affinity chromatography
PCA: principal component analysis.

## References

[1] Pan F., Malmberg R. L., and Momany M. (2007) Analysis of septins across kingdoms reveals orthology and new motifs. BMC Evol. Biol. 7, 103 17601340 10.1186/1471-2148-7-103 PMC1931588

[2] Nishihama R., Onishi M., and Pringle J. R. (2011) New insights into the phylogenetic distribution and evolutionary origins of the septins. Biol. Chem. 392, 681–687 21824002 10.1515/BC.2011.086 PMC3951473

[3] McMurray M. A., and Thorner J. (2008) Biochemical properties and supramolecular architecture of septin hetero-oligomers and septin filaments. In The Septins (Hall P. A., Russell S. E. G., and Pringle J. R., eds) pp. 49–100, John Wiley & Sons, Ltd., Chichester, West Sussex, UK

[4] Hall P. A., and Russell S. E. (2012) Mammalian septins: dynamic heteromers with roles in cellular morphogenesis and compartmentalization. J. Pathol. 226, 287–299 21990096 10.1002/path.3024

[5] Fung K. Y., Dai L., and Trimble W. S. (2014) Cell and molecular biology of septins. Int. Rev. Cell Mol. Biol. 310, 289–339 24725429 10.1016/B978-0-12-800180-6.00007-4

[6] Pringle J. R. (2008) Origins and development of the septin field. In The Septins (Hall P. A., Russell S. E. H., and Pringle J. R., eds) pp. 7–34, John Wiley & Sons, Ltd., Chichester, West Sussex, UK

[7] Weirich C. S., Erzberger J. P., and Barral Y. (2008) The septin family of GTPases: architecture and dynamics. Nat. Rev. Mol. Cell Biol. 9, 478–489 18478031 10.1038/nrm2407

[8] Hartwell L. H. (1971) Genetic control of the cell division cycle in yeast: IV. genes controlling bud emergence and cytokinesis. Exp. Cell Res. 69, 265–276 4950437 10.1016/0014-4827(71)90223-0

[9] Hartwell L. H., Culotti J., Pringle J. R., and Reid B. J. (1974) Genetic control of the cell division cycle in yeast. Science 183, 46–51 4587263 10.1126/science.183.4120.46

[10] Carroll C. W., Altman R., Schieltz D., Yates J. R., and Kellogg D. (1998) The septins are required for the mitosis-specific activation of the Gin4 kinase. J. Cell Biol. 143, 709–717 9813092 10.1083/jcb.143.3.709 PMC2148151

[11] Mino A., Tanaka K., Kamei T., Umikawa M., Fujiwara T., and Takai Y. (1998) Shs1p: a novel member of septin that interacts with spa2p, involved in polarized growth in *Saccharomyces cerevisiae*. Biochem. Biophys. Res. Commun. 251, 732–736 9790978 10.1006/bbrc.1998.9541

[12] Byers B., and Goetsch L. (1976) A highly ordered ring of membrane-associated filaments in budding yeast. J. Cell Biol. 69, 717–721 773946 10.1083/jcb.69.3.717 PMC2109696

[13] Haarer B. K., and Pringle J. R. (1987) Immunofluorescence localization of the *Saccharomyces cerevisiae CDC12* gene product to the vicinity of the 10-nm filaments in the mother-bud neck. Mol. Cell. Biol. 7, 3678–3687 3316985 10.1128/mcb.7.10.3678-3687.1987 PMC368023

[14] Ford S. K., and Pringle J. R. (1991) Cellular morphogenesis in the *Saccharomyces cerevisiae* cell cycle: localization of the *CDC11* gene product and the timing of events at the budding site. Dev. Genet. 12, 281–292 1934633 10.1002/dvg.1020120405

[15] Kim H. B., Haarer B. K., and Pringle J. R. (1991) Cellular morphogenesis in the *Saccharomyces cerevisiae* cell cycle: localization of the *CDC3* gene product and the timing of events at the budding site. J. Cell Biol. 112, 535–544 1993729 10.1083/jcb.112.4.535 PMC2288849

[16] Cid V. J., Adamíková L., Cenamor R., Molina M., Sánchez M., and Nombela C. (1998) Cell integrity and morphogenesis in a budding yeast septin mutant. Microbiology 144, 3463–3474 9884239 10.1099/00221287-144-12-3463

[17] Shulewitz M. J., Inouye C. J., and Thorner J. (1999) Hsl7 localizes to a septin ring and serves as an adapter in a regulatory pathway that relieves tyrosine phosphorylation of Cdc28 protein kinase in *Saccharomyces cerevisiae*. Mol. Cell. Biol. 19, 7123–7137 10490648 10.1128/mcb.19.10.7123 PMC84706

[18] Frazier J. A., Wong M. L., Longtine M. S., Pringle J. R., Mann M., Mitchison T. J., and Field C. (1998) Polymerization of purified yeast septins: evidence that organized filament arrays may not be required for septin function. J. Cell Biol. 143, 737–749 9813094 10.1083/jcb.143.3.737 PMC2148147

[19] Versele M., and Thorner J. (2004) Septin collar formation in budding yeast requires GTP binding and direct phosphorylation by the PAK, Cla4. J. Cell Biol. 164, 701–715 14993234 10.1083/jcb.200312070 PMC2172161

[20] Versele M., Gullbrand B., Shulewitz M. J., Cid V. J., Bahmanyar S., Chen R. E., Barth P., Alber T., and Thorner J. (2004) Protein-protein interactions governing septin heteropentamer assembly and septin filament organization in *Saccharomyces cerevisiae*. Mol. Biol. Cell 15, 4568–4583 15282341 10.1091/mbc.E04-04-0330 PMC519150

[21] Farkasovsky M., Herter P., Voss B., and Wittinghofer A. (2005) Nucleotide binding and filament assembly of recombinant yeast septin complexes. Biol. Chem. 386, 643–656 16207085 10.1515/BC.2005.075

[22] Bertin A., McMurray M. A., Grob P., Park S.-S., Garcia G. 3rd, Patanwala I., Ng H.-L., Alber T., Thorner J., Nogales E. (2008) *Saccharomyces cerevisiae* septins: supramolecular organization of hetero-oligomers and the mechanism of filament assembly. Proc. Natl. Acad. Sci. U.S.A. 105, 8274–8279 18550837 10.1073/pnas.0803330105 PMC2426963

[23] Bertin A., McMurray M. A., Pierson J., Thai L., McDonald K. L., Zehr E. A., García G. 3rd, Peters P., Thorner J., and Nogales E. (2012) Three-dimensional ultrastructure of the septin filament network in *Saccharomyces cerevisiae*. Mol. Biol. Cell 23, 423–432 22160597 10.1091/mbc.E11-10-0850 PMC3268722

[24] Ong K., Wloka C., Okada S., Svitkina T., and Bi E. (2014) Architecture and dynamic remodelling of the septin cytoskeleton during the cell cycle. Nat. Commun. 5, 5698 25474997 10.1038/ncomms6698 PMC4258872

[25] Garcia G. 3rd, Bertin A., Li Z., Song Y., McMurray M. A., Thorner J., and Nogales E. (2011) Subunit-dependent modulation of septin assembly: budding yeast septin Shs1 promotes ring and gauze formation. J. Cell Biol. 195, 993–1004 22144691 10.1083/jcb.201107123 PMC3241732

[26] Kaplan C., Jing B., Winterflood C. M., Bridges A. A., Occhipinti P., Schmied J., Grinhagens S., Gronemeyer T., Tinnefeld P., Gladfelter A. S., Ries J., and Ewers H. (2015) The absolute arrangement of subunits in cytoskeletal septin filaments in cells measured by fluorescence microscopy. Nano Lett. 15, 3859–3864 25939363 10.1021/acs.nanolett.5b00693 PMC13093486

[27] Takizawa P. A., DeRisi J. L., Wilhelm J. E., and Vale R. D. (2000) Plasma membrane compartmentalization in yeast by messenger RNA transport and a septin diffusion barrier. Science 290, 341–344 11030653 10.1126/science.290.5490.341

[28] Caudron F., and Barral Y. (2009) Septins and the lateral compartmentalization of eukaryotic membranes. Dev. Cell 16, 493–506 19386259 10.1016/j.devcel.2009.04.003

[29] McMurray M. A., and Thorner J. (2009) Septins: molecular partitioning and the generation of cellular asymmetry. Cell Div. 4, 18.1–18.14 19709431 10.1186/1747-1028-4-18 PMC2749018

[30] Oh Y., and Bi E. (2011) Septin structure and function in yeast and beyond. Trends Cell Biol. 21, 141–148 21177106 10.1016/j.tcb.2010.11.006 PMC3073566

[31] Tanaka-Takiguchi Y., Kinoshita M., and Takiguchi K. (2009) Septin-mediated uniform bracing of phospholipid membranes. Curr. Biol. 19, 140–145 19167227 10.1016/j.cub.2008.12.030

[32] Mavrakis M., Azou-Gros Y., Tsai F. C., Alvarado J., Bertin A., Iv F., Kress A., Brasselet S., Koenderink G. H., and Lecuit T. (2014) Septins promote F-actin ring formation by crosslinking actin filaments into curved bundles. Nat. Cell Biol. 16, 322–334 24633326 10.1038/ncb2921

[33] Bridges A. A., and Gladfelter A. S. (2015) Septin form and function at the cell cortex. J. Biol. Chem. 290, 17173–17180 25957401 10.1074/jbc.R114.634444 PMC4498057

[34] Kim M. S., Froese C. D., Estey M. P., and Trimble W. S. (2011) SEPT9 occupies the terminal positions in septin octamers and mediates polymerization-dependent functions in abscission. J. Cell Biol. 195, 815–826 22123865 10.1083/jcb.201106131 PMC3257574

[35] Sellin M. E., Sandblad L., Stenmark S., and Gullberg M. (2011) Deciphering the rules governing assembly order of mammalian septin complexes. Mol. Biol. Cell 22, 3152–3164 21737677 10.1091/mbc.E11-03-0253 PMC3164462

[36] Zent E., Vetter I., and Wittinghofer A. (2011) Structural and biochemical properties of Sept7, a unique septin required for filament formation. Biol. Chem. 392, 791–797 21824007 10.1515/BC.2011.082

[37] Macedo J. N., Valadares N. F., Marques I. A., Ferreira F. M., Damalio J. C., Pereira H. M., Garratt R. C., Araujo A. P. (2013) The structure and properties of septin 3: a possible missing link in septin filament formation. Biochem. J. 450, 95–105 23163726 10.1042/BJ20120851

[38] Zeraik A. E., Pereira H. M., Santos Y. V., Brandão-Neto J., Spoerner M., Santos M. S., Colnago L. A., Garratt R. C., Araújo A. P., and DeMarco R. (2014) Crystal structure of a *Schistosoma mansoni* septin reveals the phenomenon of strand slippage in septins dependent on the nature of the bound nucleotide. J. Biol. Chem. 289, 7799–7811 24464615 10.1074/jbc.M113.525352 PMC3953292

[39] Sirajuddin M., Farkasovsky M., Hauer F., Kühlmann D., Macara I. G., Weyand M., Stark H., and Wittinghofer A. (2007) Structural insight into filament formation by mammalian septins. Nature 449, 311–315 17637674 10.1038/nature06052

[40] Sirajuddin M., Farkasovsky M., Zent E., and Wittinghofer A. (2009) GTP-induced conformational changes in septins and implications for function. Proc. Natl. Acad. Sci. U.S.A. 106, 16592–16597 19805342 10.1073/pnas.0902858106 PMC2757862

[41] Zent E., and Wittinghofer A. (2014) Human septin isoforms and the GDP-GTP cycle. Biol. Chem. 395, 169–180 24246286 10.1515/hsz-2013-0268

[42] Barth P., Schoeffler A., and Alber T. (2008) Targeting metastable coiled-coil domains by computational design. J. Am. Chem. Soc. 130, 12038–12044 18698842 10.1021/ja802447e

[43] Bridges A. A., Zhang H., Mehta S. B., Occhipinti P., Tani T., and Gladfelter A. S. (2014) Septin assemblies form by diffusion-driven annealing on membranes. Proc. Natl. Acad. Sci. U.S.A. 111, 2146–2151 24469790 10.1073/pnas.1314138111 PMC3926015

[44] Renz C., Johnsson N., and Gronemeyer T. (2013) An efficient protocol for the purification and labeling of entire yeast septin rods from *E. coli* for quantitative *in vitro* experimentation. BMC Biotechnol. 13, 60.1–60.8 23889817 10.1186/1472-6750-13-60 PMC3765318

[45] Bonne D., Heuséle C., Simon C., and Pantaloni D. (1985) 4′,6-Diamidino-2-phenylindole, a fluorescent probe for tubulin and microtubules. J. Biol. Chem. 260, 2819–2825 3972806

[46] Sackett D. L., Knutson J. R., and Wolff J. (1990) Hydrophobic surfaces of tubulin probed by time-resolved and steady-state fluorescence of nile red. J. Biol. Chem. 265, 14899–14906 2394705

[47] Cooper J. A., Walker S. B., and Pollard T. D. (1983) Pyrene actin: documentation of the validity of a sensitive assay for actin polymerization. J. Muscle Res. Cell Motil. 4, 253–262 6863518 10.1007/BF00712034

[48] Hansen S. D., Zuchero J. B., and Mullins R. D. (2013) Cytoplasmic actin: purification and single molecule assembly assays. Methods Mol. Biol. 1046, 145–170 23868587 10.1007/978-1-62703-538-5_9 PMC4013826

[49] Bolanos-Garcia V. M., and Davies O. R. (2006) Structural analysis and classification of native proteins from E. coli commonly co-purified by immobilised metal affinity chromatography. Biochim. Biophys. Acta 1760, 1304–1313 16814929 10.1016/j.bbagen.2006.03.027

[50] de Val N., McMurray M. A., Lam L. H., Hsiung C. C., Bertin A., Nogales E., and Thorner J. (2013) Native cysteine residues are dispensable for the structure and function of all five yeast mitotic septins. Proteins 81, 1964–1979 23775754 10.1002/prot.24345 PMC3880206

[51] Wang W., and Malcolm B. A. (1999) Two-stage PCR protocol allowing introduction of multiple mutations, deletions and insertions using QuikChange^TM^ site-directed mutagenesis. BioTechniques 26, 680–682 10343905 10.2144/99264st03

[52] Li M. Z., and Elledge S. J. (2012) SLIC: a method for sequence- and ligation-independent cloning. Methods Mol. Biol. 852, 51–59 22328425 10.1007/978-1-61779-564-0_5

[53] Smith P. K., Krohn R. I., Hermanson G. T., Mallia A. K., Gartner F. H., Provenzano M. D., Fujimoto E. K., Goeke N. M., Olson B. J., and Klenk D. C. (1985) Measurement of protein using bicinchoninic acid. Anal. Biochem. 150, 76–85 3843705 10.1016/0003-2697(85)90442-7

[54] Al-Soufi W., Novo M., Mosquera M., and Rodríguez-Prieto F. (2009) Principal component global analysis of series of fluorescence spectra. in reviews in Fluorescence (Geddes C.D., ed) pp. 23–45, Springer-Verlag, New York Inc., New York

[55] Song Y., Madahar V., and Liao J. (2011) Development of FRET assay into quantitative and high-throughput screening technology platforms for protein-protein interactions. Ann. Biomed. Eng. 39, 1224–1234 21174150 10.1007/s10439-010-0225-x PMC3069323

[56] Hieb A. R., D'Arcy S., Kramer M. A., White A. E., and Luger K. (2012) Fluorescence strategies for high-throughput quantification of protein interactions. Nucleic Acids Res.40, e33.1-e33.13 22121211 10.1093/nar/gkr1045 PMC3299996

[57] Zeug A., Woehler A., Neher E., and Ponimaskin E. G. (2012) Quantitative intensity-based FRET approaches: a comparative snapshot. Biophys. J. 103, 1821–1827 23199910 10.1016/j.bpj.2012.09.031 PMC3491707

[58] Kelley L. A., Mezulis S., Yates C. M., Wass M. N., and Sternberg M. J. (2015) The Phyre2 web portal for protein modeling, prediction and analysis. Nat. Protoc. 10, 845–858 25950237 10.1038/nprot.2015.053 PMC5298202

[59] Bertin A., McMurray M. A., Thai L., Garcia G. 3rd, Votin V., Grob P., Allyn T., Thorner J., and Nogales E. (2010) Phosphatidylinositol-4,5-bisphosphate promotes budding yeast septin filament assembly and organization. J. Mol. Biol. 404, 711–731 20951708 10.1016/j.jmb.2010.10.002 PMC3005623

[60] Kenworthy A. K. (2001) Imaging protein-protein interactions using fluorescence resonance energy transfer microscopy. Methods 24, 289–296 11403577 10.1006/meth.2001.1189

[61] McMurray M. A., and Thorner J. (2008) Septin stability and recycling during dynamic structural transitions in cell division and development. Curr. Biol. 18, 1203–1208 18701287 10.1016/j.cub.2008.07.020 PMC2562167

[62] Lakowicz J. R. (2006) Principles of Fluorescence Spectroscopy, 3rd Ed., Springer-Verlag, New York Inc., New York

[63] McMurray M. A., Bertin A., Garcia G. 3rd, Lam L., Nogales E., and Thorner J. (2011) Septin filament formation is essential in budding yeast. Dev. Cell 20, 540–549 21497764 10.1016/j.devcel.2011.02.004 PMC3079881

[64] Finnigan G. C., Takagi J., Cho C., and Thorner J. (2015) Comprehensive genetic analysis of paralogous terminal septin subunits Shs1 and Cdc11 in *Saccharomyces cerevisiae*. Genetics 200, 821–841 25971665 10.1534/genetics.115.176495 PMC4512546

[65] Finnigan G. C., Booth E. A., Duvalyan A., Liao E. N., and Thorner J. (2015) The carboxy-terminal tails of septins Cdc11 and Shs1 recruit myosin-II binding factor Bni5 to the bud neck in *Saccharomyces cerevisiae*. Genetics 200, 843–862 25971666 10.1534/genetics.115.176503 PMC4512547

[66] Rodal A. A., Kozubowski L., Goode B. L., Drubin D. G., and Hartwig J. H. (2005) Actin and septin ultrastructures at the budding yeast cell cortex. Mol. Biol. Cell 16, 372–384 15525671 10.1091/mbc.E04-08-0734 PMC539180

[67] de Almeida Marques I., Valadares N. F., Garcia W., Damalio J. C., Macedo J. N., de Araújo A. P., Botello C. A., Andreu J. M., and Garratt R. C. (2012) Septin C-terminal domain interactions: implications for filament stability and assembly. Cell Biochem. Biophys. 62, 317–328 22001952 10.1007/s12013-011-9307-0

[68] Zhang J., Kong C., Xie H., McPherson P. S., Grinstein S., and Trimble W. S. (1999) Phosphatidylinositol polyphosphate binding to the mammalian septin H5 is modulated by GTP. Curr. Biol. 9, 1458–1467 10607590 10.1016/s0960-9822(00)80115-3

[69] Lee P. R., Song S., Ro H. S., Park C. J., Lippincott J., Li R., Pringle J. R., De Virgilio C., Longtine M. S., and Lee K. S. (2002) Bni5p, a septin-interacting protein, is required for normal septin function and cytokinesis in *Saccharomyces cerevisiae*. Mol. Cell. Biol. 22, 6906–6920 12215547 10.1128/MCB.22.19.6906-6920.2002 PMC134035

[70] Fang X., Luo J., Nishihama R., Wloka C., Dravis C., Travaglia M., Iwase M., Vallen E. A., and Bi E. (2010) Biphasic targeting and cleavage furrow ingression directed by the tail of a myosin II. J. Cell Biol. 191, 1333–1350 21173112 10.1083/jcb.201005134 PMC3010076

[71] Schneider C., Grois J., Renz C., Gronemeyer T., and Johnsson N. (2013) Septin rings act as a template for myosin higher-order structures and inhibit redundant polarity establishment. J. Cell Sci. 126, 3390–3400 23750004 10.1242/jcs.125302

[72] Pollard T. D., and Cooper J. A. (2009) Actin, a central player in cell shape and movement. Science 326, 1208–1212 19965462 10.1126/science.1175862 PMC3677050

[73] Iwase M., Luo J., Bi E., and Toh-e A. (2007) Shs1 plays separable roles in septin organization and cytokinesis in *Saccharomyces cerevisiae*. Genetics 177, 215–229 17603111 10.1534/genetics.107.073007 PMC2013704

[74] Buttery S. M., Kono K., Stokasimov E., and Pellman D. (2012) Regulation of the formin Bnr1 by septins and a MARK/Par1-family septin-associated kinase. Mol. Biol. Cell 23, 4041–4053 22918953 10.1091/mbc.E12-05-0395 PMC3469519

[75] Miller D. J. (2004) Sydney Ringer; physiological saline, calcium and the contraction of the heart. J. Physiol. 555, 585–587 14742734 10.1113/jphysiol.2004.060731 PMC1664856

[76] Dobbelaere J., Gentry M. S., Hallberg R. L., and Barral Y. (2003) Phosphorylation-dependent regulation of septin dynamics during the cell cycle. Dev. Cell 4, 345–357 12636916 10.1016/s1534-5807(03)00061-3

[77] Egelhofer T. A., Villén J., McCusker D., Gygi S. P., and Kellogg D. R. (2008) The septins function in G_1_ pathways that influence the pattern of cell growth in budding yeast. PLoS One 3, e2022.2021–e2022.2014 18431499 10.1371/journal.pone.0002022 PMC2291192

[78] Gladfelter A. S., Pringle J. R., and Lew D. J. (2001) The septin cortex at the yeast mother-bud neck. Curr. Opin. Microbiol. 4, 681–689 11731320 10.1016/s1369-5274(01)00269-7

[79] Spiliotis E. T., and Nelson W. J. (2006) Here come the septins: novel polymers that coordinate intracellular functions and organization. J. Cell Sci. 119, 4–10 16371649 10.1242/jcs.02746 PMC3368708

