# Supplementary material for: A Förster Resonance Energy Transfer (FRET)-based System Provides Insight into the Ordered Assembly of Yeast Septin Hetero-octamers
Source: J Biol Chem. 2015 Sep 28;290(47):28388–401. doi: 10.1074/jbc.M115.683128 (PMC4653696; doi:10.1074/jbc.M115.683128)
Supplement: Supplemental Data [file supp_290_47_28388__index.html]

A Foerster Resonance Energy Transfer (FRET)-based System Provides Insight into the Ordered Assembly of Yeast Septin Hetero-octamers — A Förster Resonance Energy Transfer (FRET)-based System Provides Insight into the Ordered Assembly of Yeast Septin Hetero-octamers — A Spectroscopic Approach for Analysis of Septin Assembly — Supplemental Data 

# A Förster Resonance Energy Transfer (FRET)-based System Provides Insight into the Ordered Assembly of Yeast Septin Hetero-octamers

## Supplemental Data

- Supplemental Data (.pdf, 8.4 MB) - Contains extensive multi-panel (up to 12-15 separate images) of fluorescent micrographs.
