# Supplementary material for: A Förster Resonance Energy Transfer (FRET)-based System Provides Insight into the Ordered Assembly of Yeast Septin Hetero-octamers
Source: J Biol Chem. 2015 Sep 28;290(47):28388–401. doi: 10.1074/jbc.M115.683128 (PMC4653696; doi:10.1074/jbc.M115.683128)
Supplement: Supplemental Data [file 10.1074_M115.683128_jbc.M115.683128-1.pdf]

SUPPLEMENTAL INFORMATION

A FRET-based System Provides Insight into the Ordered Assembly of Yeast Septin Hetero-octamers

Elizabeth A. Booth[1a], Eleanor W. Vane[1b], Dustin Dovala[2c] and Jeremy Thorner[1*]

[1]Division of Biochemistry, Biophysics, and Structural Biology, Department of Molecular and Cell Biology, University of California, Berkeley, CA 94720-3202
[2]Program in Microbial Pathogenesis and Host Defense, Department of Microbiology and Immunology, University of California School of Medicine, San Francisco, CA 94158-2200

Running title**:** *A spectroscopic approach for analysis of septin assembly*

***To whom correspondence should be addressed: Prof. Jeremy Thorner, Dept. of Molecular and Cell Biology, University of California, Rm. 526, Barker Hall, Berkeley, CA 94720-3202. Phone: (510) 642-2558, FAX: (510) 642- 6420, E-mail: jthorner@berkeley.edu

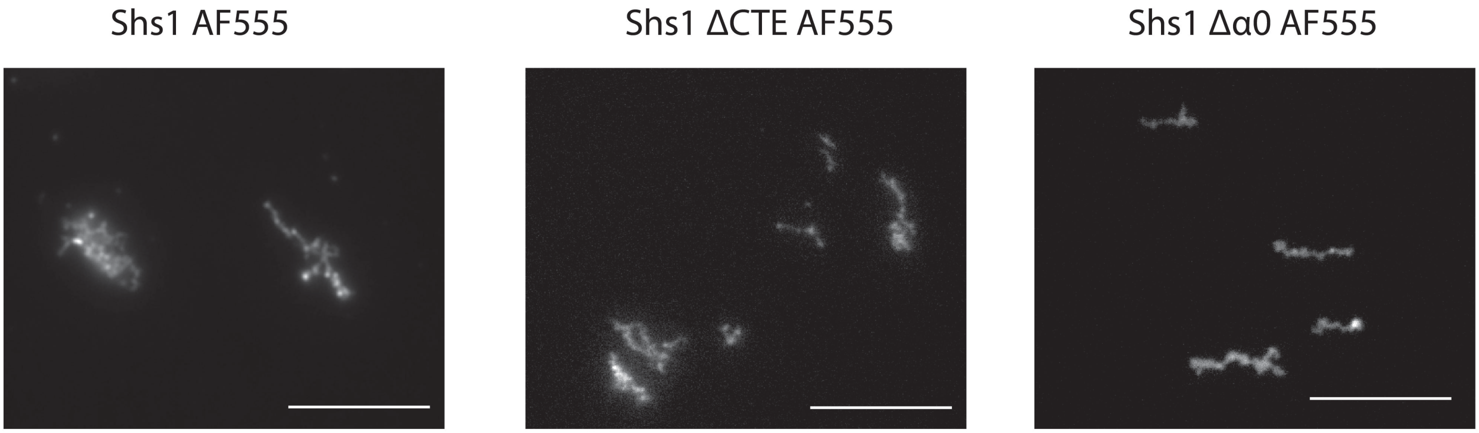

Figure S1: Micrographs of assembled Shs1 septins. Shs1^{AF555} constructs self-assembled for 1 hour at room temperature before plating on PLL coated slides.

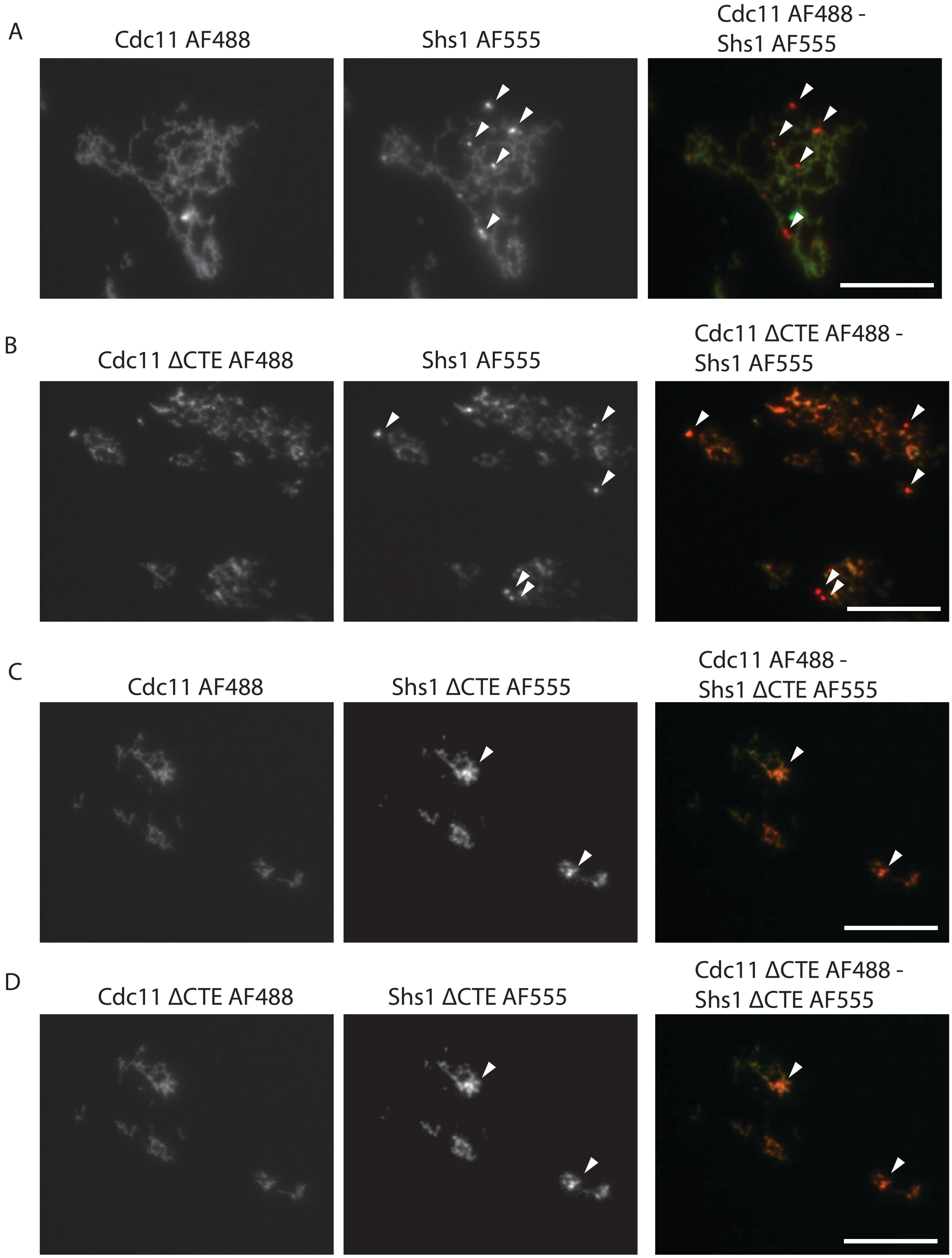

Figure S2: Micrographs of assembled Cdc11 and Shs1 septins with CTE deletions. Equimolar concentrations for $Cdc11^{AF488}$ septins and $Shs1^{AF555}$ septins self-assembled for 1 hour at room temperature before plating on PLL coated slides. (A-D) Leftmost column shows the $Cdc11^{AF488}$ construct. The center column shows the $Shs1^{AF555}$ construct. The rightmost column shows the overlay of the AF555 and AF488 labeled constructs. Arrows indicate the formation of Shs1 puncta.

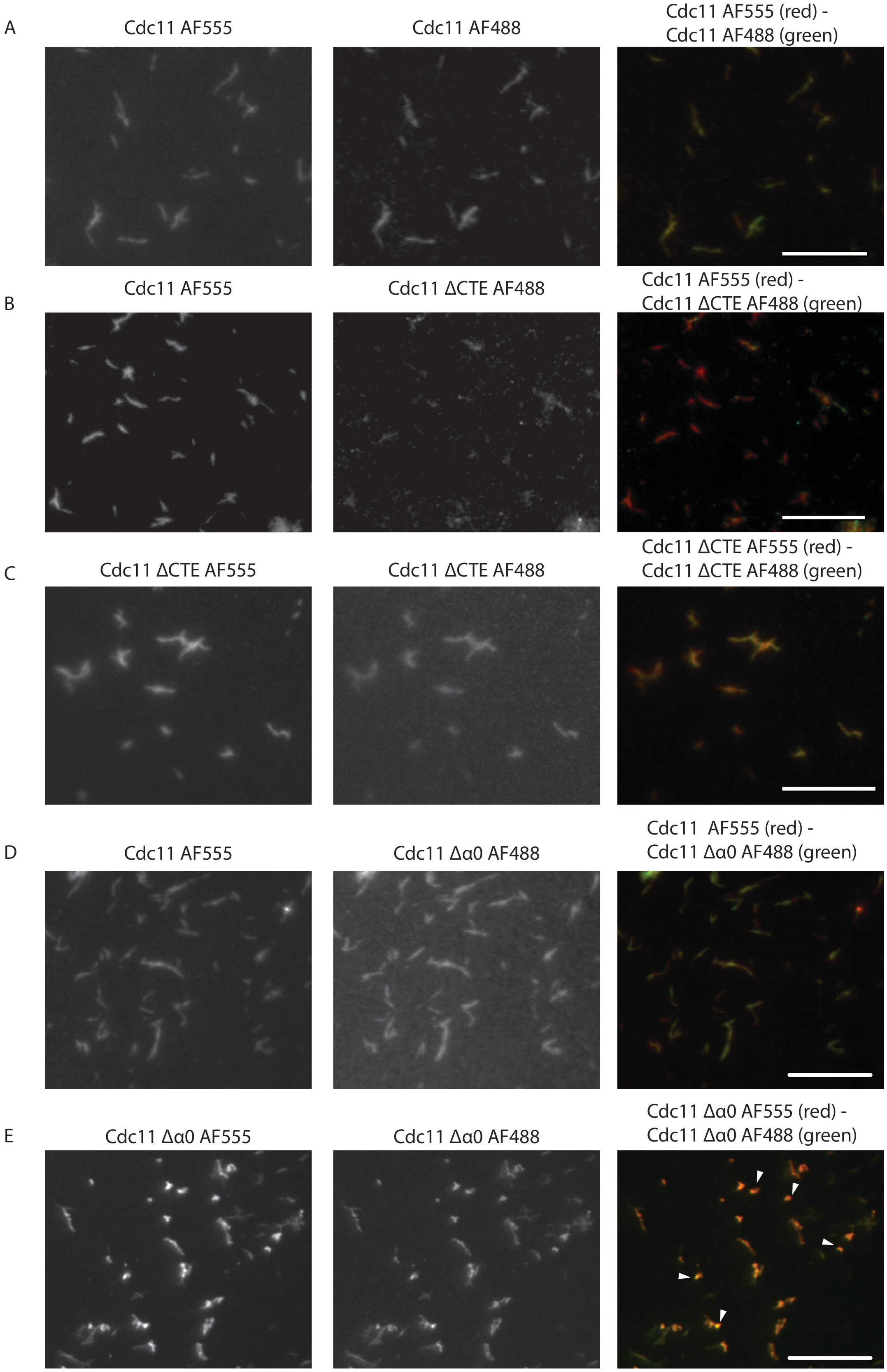

Figure S3: Micrographs of assembled Cdc11 septins. Equimolar concentrations for AF488 and AF555 labeled septins self-assembled for 1 hour at room temperature before plating on PLL coated slides. (A-E) Leftmost column shows the $Cdc11^{AF555}$ construct. The center column shows the $Cdc11^{AF488}$ construct. The rightmost column shows the overlay of the AF555 and AF488 labeled constructs.

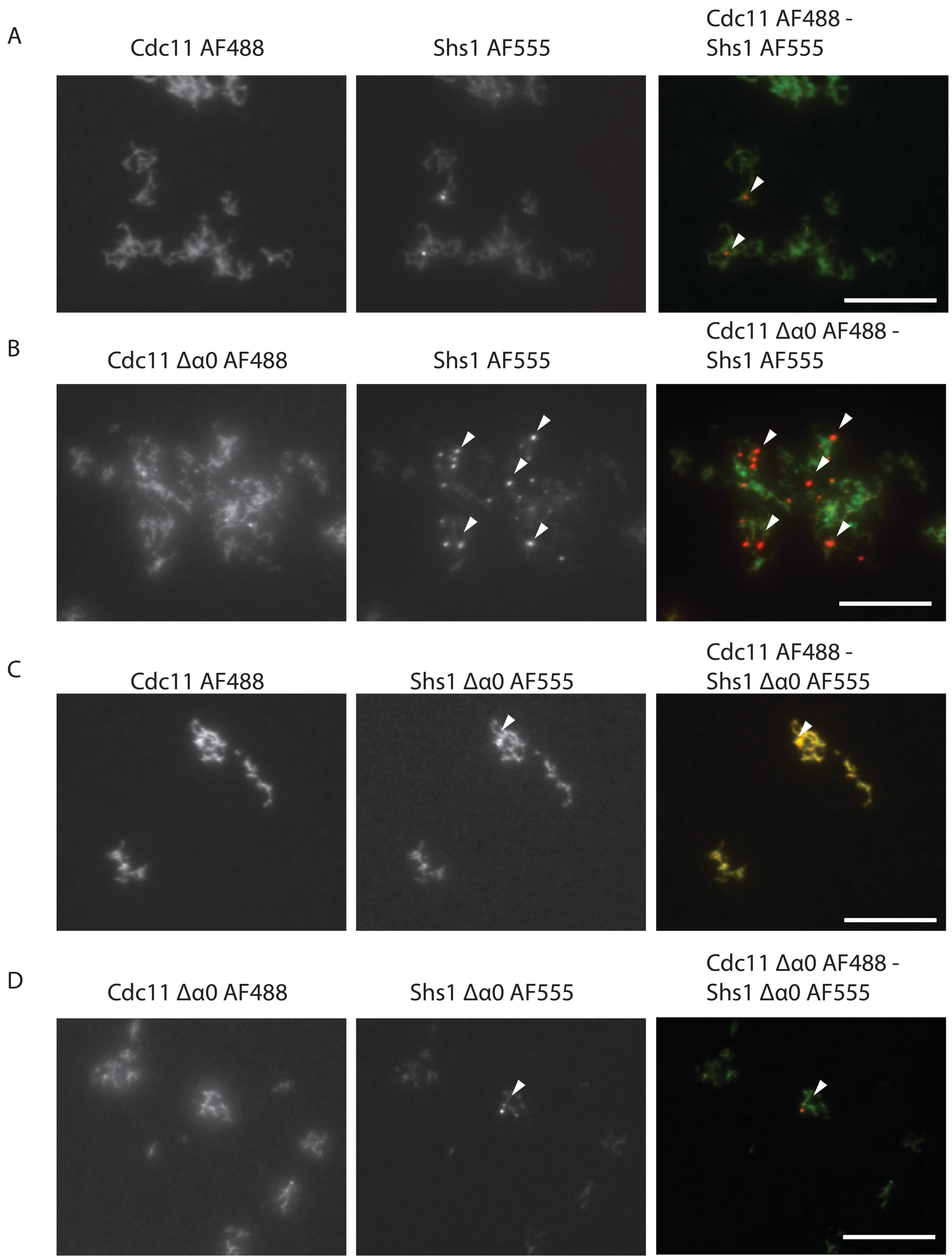

Figure S4: Micrographs of assembled Cdc11 and Shs1 septins with α0 deletions. Equimolar concentrations for Cdc11^{AF488} septins and Shs1^{AF555} septins self-assembled for 1 hour at room temperature before plating on PLL coated slides. (A-D) Leftmost column shows the Cdc11^{AF488} construct. The center column shows the Shs1^{Af555} construct. The rightmost column shows the overlay of the AF555 and AF488 labeled constructs. Arrows indicate the formation of Shs1 puncta.
